# Cattle Sex-Specific Recombination and Genetic Control from a Large Pedigree Analysis

**DOI:** 10.1371/journal.pgen.1005387

**Published:** 2015-11-05

**Authors:** Li Ma, Jeffrey R. O'Connell, Paul M. VanRaden, Botong Shen, Abinash Padhi, Chuanyu Sun, Derek M. Bickhart, John B. Cole, Daniel J. Null, George E. Liu, Yang Da, George R. Wiggans

**Affiliations:** 1 Department of Animal and Avian Sciences, University of Maryland, College Park, Maryland, United States of America; 2 Department of Medicine, University of Maryland Medical School, Baltimore, Maryland, United States of America; 3 Animal Genomics and Improvement Laboratory, Agricultural Research Service, United States Department of Agriculture (USDA), Beltsville, Maryland, United States of America; 4 National Association of Animal Breeders, Columbia, Missouri, United States of America; 5 Department of Animal Science, University of Minnesota, St. Paul, Minnesota, United States of America; Albert Einstein College of Medicine, UNITED STATES

## Abstract

Meiotic recombination is an essential biological process that generates genetic diversity and ensures proper segregation of chromosomes during meiosis. From a large USDA dairy cattle pedigree with over half a million genotyped animals, we extracted 186,927 three-generation families, identified over 8.5 million maternal and paternal recombination events, and constructed sex-specific recombination maps for 59,309 autosomal SNPs. The recombination map spans for 25.5 Morgans in males and 23.2 Morgans in females, for a total studied region of 2,516 Mb (986 kb/cM in males and 1,085 kb/cM in females). The male map is 10% longer than the female map and the sex difference is most pronounced in the subtelomeric regions. We identified 1,792 male and 1,885 female putative recombination hotspots, with 720 hotspots shared between sexes. These hotspots encompass 3% of the genome but account for 25% of the genome-wide recombination events in both sexes. During the past forty years, males showed a decreasing trend in recombination rate that coincided with the artificial selection for milk production. Sex-specific GWAS analyses identified *PRDM9* and *CPLX1* to have significant effects on genome-wide recombination rate in both sexes. Two novel loci, *NEK9* and *REC114*, were associated with recombination rate in both sexes, whereas three loci, *MSH4*, *SMC3* and *CEP55*, affected recombination rate in females only. Among the multiple *PRDM9* paralogues on the bovine genome, our GWAS of recombination hotspot usage together with linkage analysis identified the *PRDM9* paralogue on chromosome 1 to be associated in the U.S. Holstein data. Given the largest sample size ever reported for such studies, our results reveal new insights into the understanding of cattle and mammalian recombination.

## Author Summary

Previous studies on cattle recombination largely focused on males. Using a large Holstein sample from the USDA national database, we studied both male and female recombination by assembling paternal and maternal recombination events in at least three generations. This unique data set provides unprecedented statistical power to study cattle genome recombination in the two sexes: (1) We report for the first time that bulls have more recombination than cows, contrary to the common perception that females have more recombination than males as observed in many mammalian species including humans and mice, and that the sex difference in recombination primarily occurs near the subtelomeric regions of all bovine autosomes; (2) We identify several genes associated with cattle recombination in both females and males, and genes affecting female recombination only; (3) We define putative recombination hotspots and find the cattle *PRDM9* gene to be associated with recombination hotspot usage. These results provide new insights for understanding cattle and mammalian genome recombination.

## Introduction

In eukaryotes, meiotic recombination through reciprocal crossovers is an essential biological process that ensures the proper segregation of homologous chromosomes during meiosis [1–4]. Any mistakes or aberrations during this process can result in aneuploidy, a potentially deleterious outcome [5,6]. Mechanisms of meiotic recombination are therefore conserved, and the location and frequency of meiotic crossovers are biologically regulated [7]. In addition, meiotic recombination contributes to genetic diversity by reshuffling maternal and paternal genetic alleles into the next generation, which provides novel combinations of genetic variants to selection and evolution [8].

Considerable variation in recombination rate among individuals has been documented from pedigree-based studies in humans and mice [9,10]. In humans, several genes have been identified to be associated with individual-level variation in recombination rate, including *CPLX1*, *RNF212* and *PRDM9* [9,11–13]. Additionally, locations of recombination crossovers are not uniformly distributed along the genome, but are mainly regulated by the PRDM9 protein during the initiation of meiotic recombination [11–13]. Recombination hotspots, i.e., short chromosome regions where crossovers occur more frequently than in other regions, have been identified in humans and mice [14–16], and *PRDM9* has been found to be associated with the percentage of crossovers in hotspots that is termed as ‘hotspot usage’ [9,12]. Furthermore, a recent study reported differences in locations of double-strand breaks between different *PRDM9* alleles in humans [17]. While all these findings are restricted to humans and mice, studies in other mammalian species can provide comparative information for understanding recombination, especially in those that have the *PRDM9* homologue such as cattle.

Recombination rate varies considerably between the two sexes in many species, particularly in mammals [8,18,19]. In humans and mice, females have higher recombination rates or more crossovers than males [18,20–22]. In sheep, however, males tend to have more crossovers [23]. In cattle, previous studies have failed to find sex difference, as these studies were limited by the small to moderate sample sizes and numbers of genetic markers [24–28]. Two cattle studies on male recombination using the bovine 50K SNP chip were recently reported. Sandor et al. characterized cattle male meiotic recombination using 10,192 bulls from the Netherlands and 3783 bulls from New Zealand with 19,487 SNPs in common between the two groups. [29]. Weng et al. reported male recombination features and related genetic loci in beef cattle with a moderate sample size (2,778 Angus and 1,485 Limousin sire-offspring pairs) [30]. While these studies provided insights into male recombination in cattle, neither study had information about female recombination to provide a female recombination map. Large scale study on sex differences in genome-wide recombination including the genetic control of female recombination remains unavailable. Sandor et al. (2012) reported an association between recombination hotspot usage and *PRDM9* in bulls, but localized the gene to chromosome X. However, *PRDM9* has four paralogues in the bovine genome and previous studies have found signals of positive selection associated with the copy on chromosome 1 [31]. This study provides clear evidence that the *PRDM9* paralogue on chromosome 1 is associated with recombination hotspot usage in the U.S. Holstein population.

Cattle are a uniparous species where the population structure generally lacks female recombination information that requires at least three generations. The United States Department of Agriculture (USDA) has received genotypes for over half a million Holstein cattle spanning several generations for genomic selection. This large multi-generational structure overcomes the problem of lacking female recombination information in cattle and provides a unique opportunity to study genome recombination in both females and males with unprecedented statistical power. Utilizing this large sample, the present study seeks to comprehensively survey the sex-specific patterns of meiotic recombination and to understand the genetic basis of individual differences in recombination in males and females. We also aim to generate the first SNP-based recombination maps in the two sexes and to evaluate the trend of meiotic recombination features that might be associated with the long-term artificial selection for dairy production.

## Results

**Fig 1:**
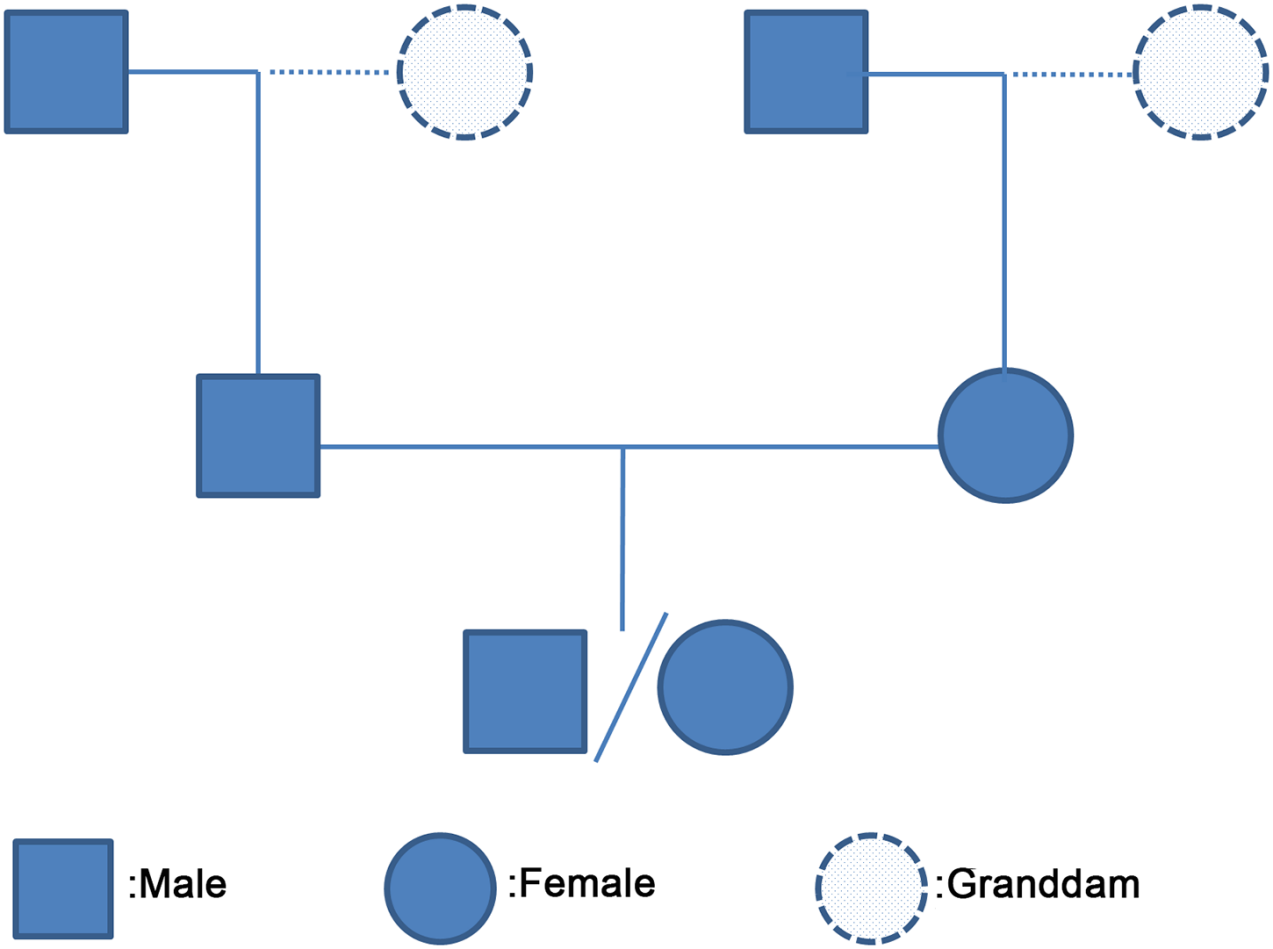
A three-generation family used for phasing haplotypes and inferring crossovers. We extracted 185,917 three-generation families, where the offspring, two parents and two grandsires were genotyped, from a large pedigree of Holstein cattle with over half million genotyped cattle maintained in USDA-AGIL (S1, S2 and S3 Tables). Depending on the number of genotyped granddams, we collected 67,690, 76,318 and 41,909 three-generation families respectively with two, one and zero genotyped granddams.

We extracted a total of 185,917 three-generation families that included one offspring, both parents, and two grandsires per family genotyped by various SNP chips ranging from 3K, 7K, to 770K SNPs from the large Holstein cattle pedigree with over half million genotyped cattle (Fig 1 and S1 Table). In each family, we phased the genotypes of the two parents and the offspring, and inferred recombination events for a paternal meiosis from the sire/offspring pair and for a maternal meiosis from the dam/offspring pair. In total, we inferred over 8.5 million paternal and maternal recombination events, which were used to estimate recombination rate between SNP intervals and individual-level recombination statistics. All the 185,917 paternal and maternal meioses were included in the GWAS of recombination rate, and only high-quality meioses from the 50K SNP data (70,715 paternal and 61,616 maternal) were used for the construction of recombination maps and GWAS of hotspot usage (S2 and S3 Tables). The sample sizes are the largest thus far available for studying cattle recombination. Even the sample size of high-quality meioses alone (paternal and maternal together) are already 13 times larger than the biggest sample size of previous cattle recombination studies [29,30]. To ensure data quality, we used the USDA Animal Genomics and Improvement Laboratory (AGIL) SNP coordinates and excluded the X chromosome from recombination calculation due to the poor quality of current genome assembly for the X chromosome [30,32,33].

### Cattle male and female recombination maps

**Fig 2:**
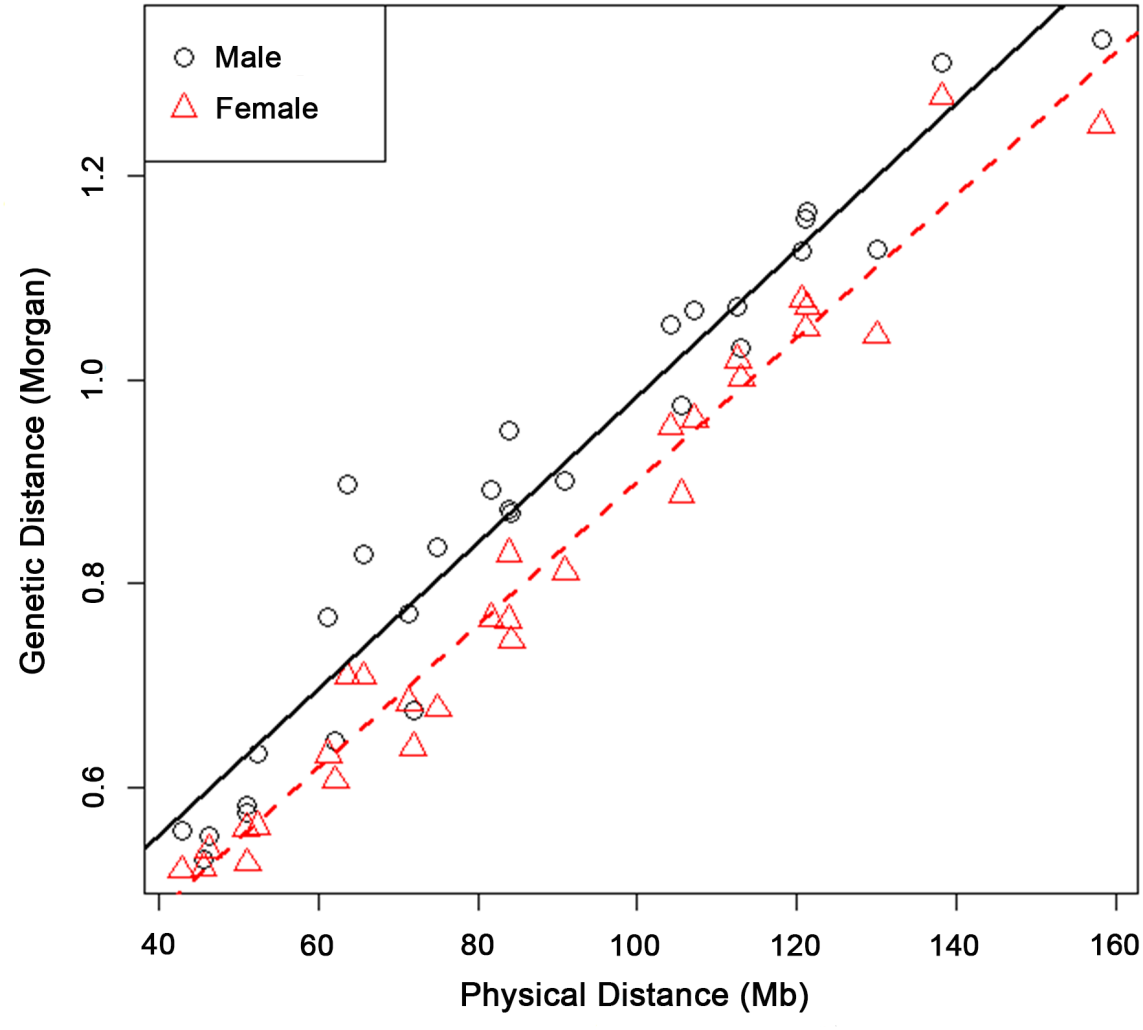
Male and female genetic lengths versus physical lengths of the 29 Bovine autosomes. A linear regression was fitted and plotted for males and females separately. Male recombination rate was higher than that of females for each of the 29 Bovine autosomes. The physical and genetic lengths of Bovine autosomes had a strong positive correlation of 0.960 in males and 0.985 in females.

The recombination map was calculated as the recombination rates between adjacent SNPs based on the AGIL SNP map. Using an EM algorithm [9], we constructed cattle sex-specific recombination maps for all bovine autosomes, spanning 25.5 Morgans in males and 23.2 Morgans in females (Supplemental File). These are the first such cattle recombination maps using genome-wide SNP markers. Also for the first time, we identified a significant sex difference in cattle recombination rate, with the male map being 2.3 Morgans (10%) longer than the female map. Moreover, the male map was longer than the female map for every chromosome, with the difference ranging from 0.007 Morgans (1.4%) for chromosome 27 to 0.188 Morgans (26.5%) for chromosome 19 (Fig 2). The male and female recombination maps were positively correlated across the SNP intervals (*R* = 0.636), which is similar to the results in humans [9]. To evaluate whether SNP information measures differ between the two sexes, we compared the distribution of the number of informative SNPs in the two sexes and found no difference (S1 Fig).

The sex-specific recombination maps comprised of 59,309 SNP markers for all 29 bovine autosomes, with an average physical distance of 42.4 kb between adjacent SNPs and an average genetic distance of 0.043 cM in males and 0.039 cM in females, respectively. The 59,309 autosomal SNPs covered a total physical length of 2,516 Mb with 986 kb/cM in males and 1,085 kb/cM in females. The physical and genetic lengths of bovine autosomes had strong positive correlations of 0.960 in males and 0.985 in females across the 29 autosomes (Fig 2). Our estimated male map size of 25.5 Morgans for autosomes was consistent with a recent study using 10,106 cattle sperms and a 50K SNP chip that had an estimated genetic map length of 25.7 Morgans [29]. To evaluate the power of detecting crossovers in our study, we conducted simulations using the same settings, including a three-generation family structure and 50K SNP chip. The result showed that the power for identifying a crossover was 97.6%. Due to the large sample size of the study, our recombination maps extended far to the two ends of the chromosomes and an obvious decline in recombination rate was observed at a distance of 2 Mb.

### Male and female recombination hotspots

**Fig 3:**
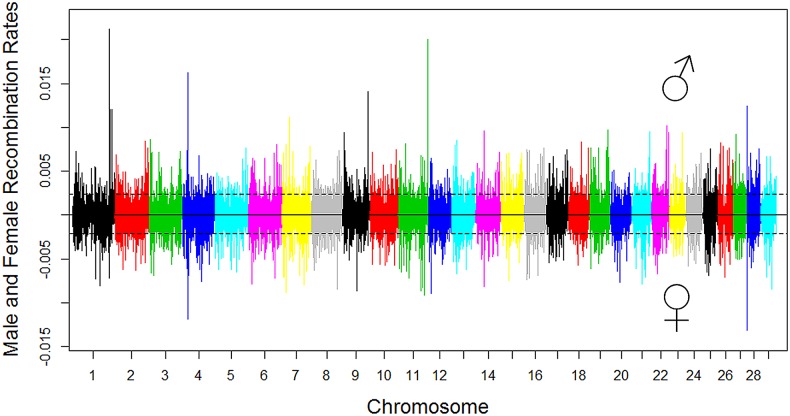
Genome-wide distribution of male and female recombination rates in SNP intervals of each chromosome. Male recombination rate was shown in the top half of the figure and female recombination rate was shown in the bottom by multiplying the original female recombination rate with ‒1. The dotted lines denote the threshold of 2.5 standard deviations plus the mean for recombination hotspot, 0.0023 for males and 0.0021 for females. Different colors were used to distinguish the 29 bovine autosomal chromosomes.

Cattle male and female recombination rates are unevenly distributed along the genome (Fig 3), consistent with the observations in humans and mice [9,11,12]. By defining hotspots as SNP intervals with recombination rate >2.5 standard deviations greater than the mean [29], we identified 1,792 hotspots for males and 1,885 hotspots for females, with 720 of them shared between sexes (i.e., 40.2% for males and 38.2% for females were shared). The difference in recombination rate in subtelomeric regions between males and females largely explains the low sharing of hotspots between the two sexes (Fig 3). The male recombination hotspots covered 3.0% of the physical length of the autosomes but accounted for 25.1% of the total male recombination events. The female hotspots comprised of 3.2% of the autosomes but accounted for 25.6% of the total recombination. The 720 shared hotspots accounted for a similar amount of the total recombination events in males (11.2%) and females (11.1%). The low sharing of hotspots between the two sexes (38.2% ~ 40.2%) could have allowed opportunity for sire selection for combined genetic material not as easily obtainable in females, noting that sire selection has been the primary genetic selection in dairy cattle and has been highly efficient.

Although our approach has a measure to minimize the effect of genotyping and genome assembly errors by requiring at least three informative markers for a crossover call, we caution that big chunks of genome assembly errors may still lead to spurious recombination hotspots [34]. To filter false-positive hotspots, we conducted pairwise linkage analysis using Locusmap [35] and checked the linkage disequilibrium (LD) pattern between each of the SNPs near a hotspot and all other SNPs on the same chromosome. As a result of this analysis, nine SNPs that showed suspicious linkage and LD patterns were removed from all analyses (S2 Fig and S4 Table). These results were consistent with the observation that many of the hotspots with recombination rate greater than 0.01 were likely due to genome assembly errors [34].

### Variation in recombination rate along the chromosome

We assessed the relationship between recombination rate and chromosomal locations, as recombination rates are known to differ considerably across chromosomal locations, including telomeres and centromeres. After removing the 2-Mb regions at the two ends of each chromosome where the power for identifying crossovers was reduced due to low SNP coverage [9], we fitted a smooth spline model of recombination rate on relative chromosomal positions, to investigate how recombination rate changes along the chromosome in each sex separately.

**Fig 4:**
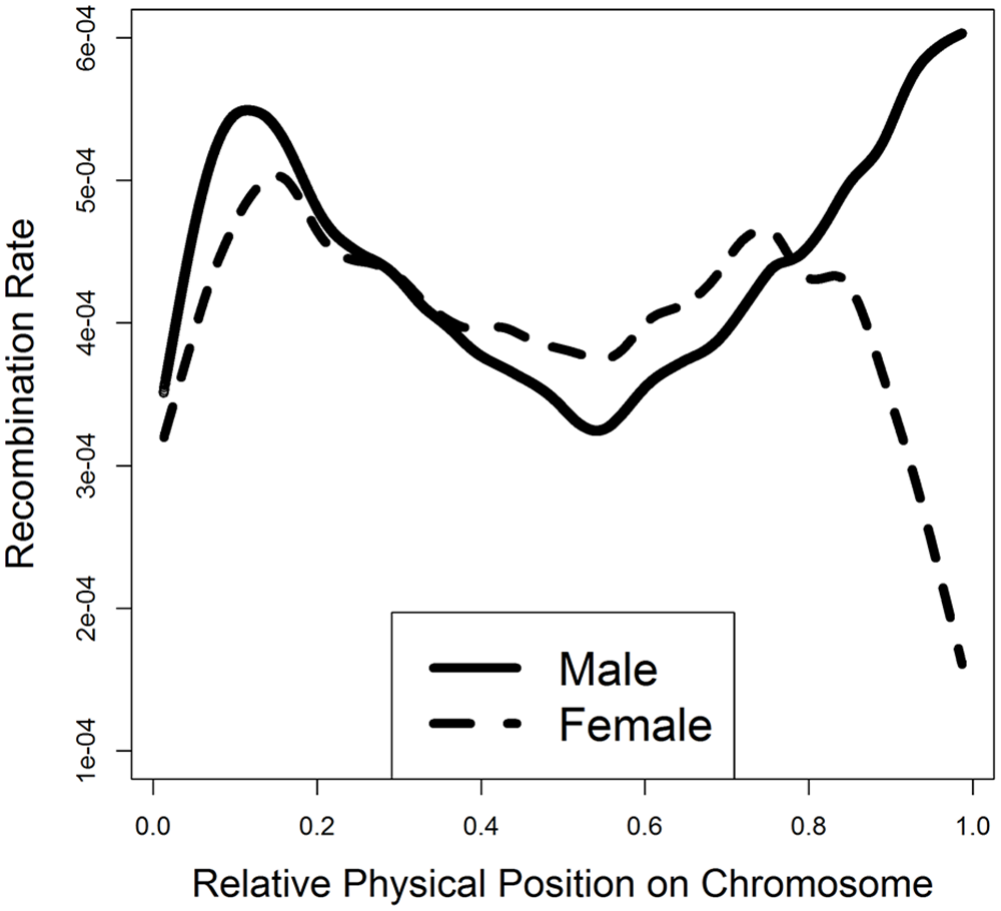
Smooth spline plotting of male and female recombination rates along a chromosome. The relative physical position for each SNP interval on a chromosome was calculated by standardizing the original physical position by the chromosome length: a value of zero corresponds to the beginning of a chromosome and a value of one corresponds to the end. The smooth spline model was fitted across all of the 29 autosomes.

All cattle autosomes are acrocentric with the centromere located at the beginning and the telomere at the end of each chromosome [36]. Males had a considerably higher recombination rate than females in the subtelomeric regions, ~15% of the chromosome to the telomeric end (Fig 4). Consistently, a male-biased recombination near telomeres was observed for each of the 29 autosomes (S3 Fig). More importantly, the subtelomeric regions accounted for all the sex differences in genome-wide recombination rate, showing a difference of 2.4 Morgans in recombination rate between males and females in the last 15% of the autosomal chromosomes near the telomere. Although a higher male recombination rate in subtelomeric regions has been shown in humans and mice [9,19,37], this is the first such report in cattle. As expected, we also observed a very low recombination rate near the centromere, the beginning of each chromosome, for both males and females. Interestingly, the middle of a chromosome had a decreased recombination rate, although the centromere is far from the middle. This low recombination rate in the middle of a chromosome was not universal across all chromosomes, but more pronounced for chromosomes 9, 10, 11, 13, 15,16, 19 and 23 (S3 Fig).

To evaluate whether crossover interference contributed to the bi-modal distribution of recombination events along the chromosome (Fig 4), we separated single-crossover and double-crossover chromosomes and then generated smooth-spline plots for recombination rate along chromosomal locations for these two sets of chromosomes separately (S4 Fig). A lower recombination rate in the middle of chromosomes for double-crossover chromosomes than single-crossover chromosomes indicates the effect of positive crossover interference, consistent with the observation in mouse [38].

### Genetic control of genome-wide recombination rate in males and females

We conducted GWAS analysis of genome-wide recombination rates for 3,224 sires and 53,125 dams separately. We corrected for the effect of SNP number so that the average number of recombination events was the same regardless of the genotyping assay used (S2 and S3 Tables). Due to the intensive use of artificial insemination, males had more progeny than females, resulting in more recombination measurements from each sires but a smaller number of sires than dams in the sample, even though the total numbers of meioses were the same between the two sexes. For each animal, the average number of recombination after correction across all meioses was used as a phenotype and the number of measurements/meioses was used as a weight. We tested the association between sex-specific genome-wide recombination rates and 310,790 imputed SNPs using a linear mixed model. We used variable residual variances that are inversely proportional to the weight and a genome-wide significance level of 1.6×10^−7^ from the Bonferroni correction.

**Fig 5:**
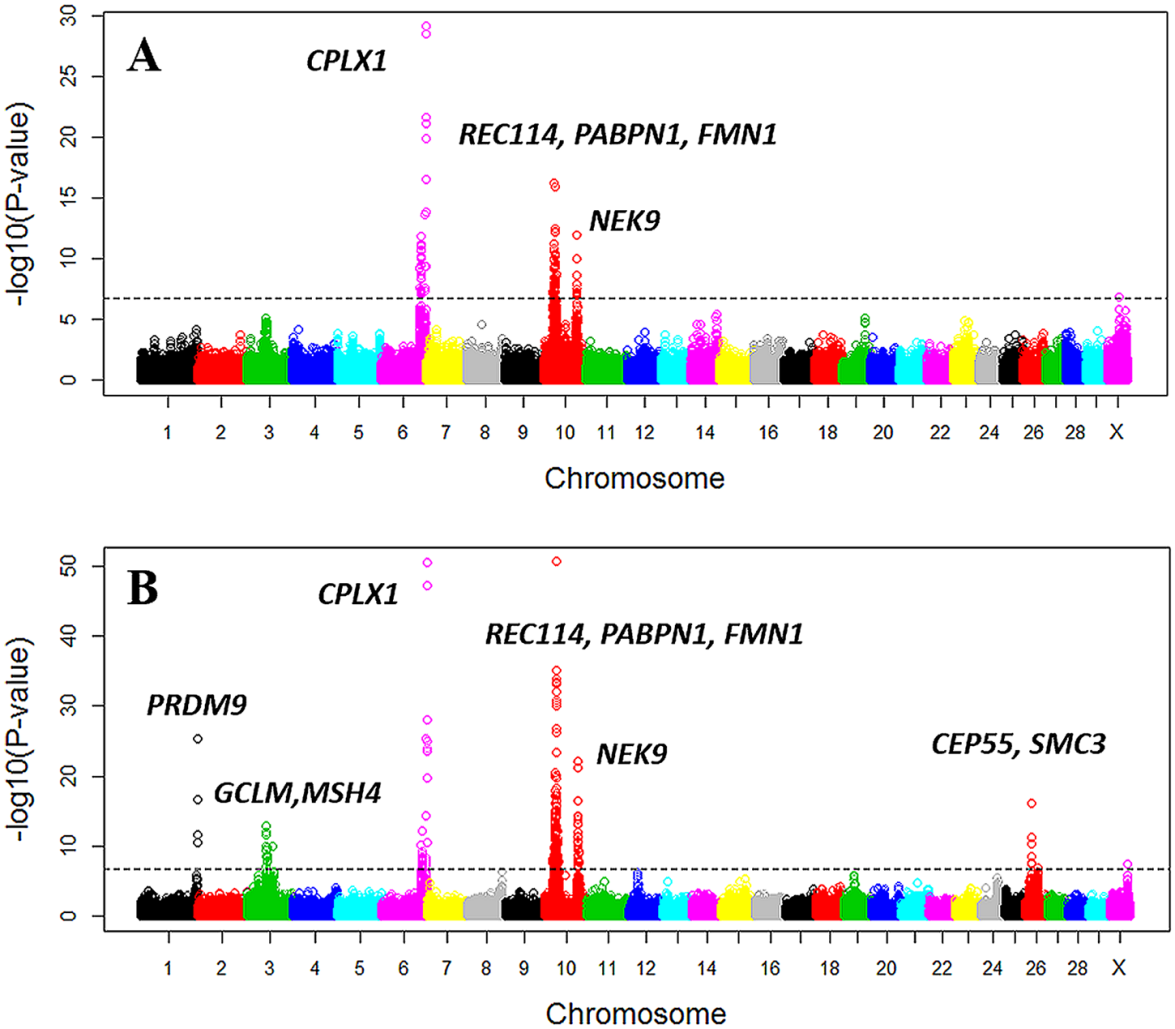
Manhattan plot of the GWAS of genome-wide recombination rates for males (A) and females (B). Different colors were used to distinguish the 29 chromosomes. The genome-wide significance level of 1.6×10^−7^ was shown by the horizontal dotted line. USDA-AGIL SNP coordinates were used for plotting, which placed *PRDM9*-linked SNPs to the end of Chromosome 1. Note that UMD3.1 assembly placed *PRDM9* near the middle of Chromosome 1.

A total of thirteen loci were identified to have significant effects on recombination rate, four loci on male recombination rate and nine loci on female recombination rate, among which three loci were shared between the two sexes (Table 1 and Fig 5 and S5 Fig). The three shared loci (one on chromosome 6 and two on chromosome 10) were among the strongest associations (Table 1 and Fig 5). The top SNP at the chromosome 6 locus, rs110253089 (*P*_female_ = 2.95×10^−51^; *P*_male_ = 7.34×10^−30^), was located in the intron of the *CPLX1* gene, which was associated with genome-wide recombination rate in humans [39]. Using this SNP as a covariate in a conditional analysis, other originally associated SNPs at the same locus were no longer significantly associated with recombination rate, suggesting a potential single underlying QTL at this locus. We found two significantly associated loci on chromosome 10. The associations at the first locus peaked at SNP rs137264867 (*P*_female_ = 2.62×10^−51^; *P*_male_ = 1.07×10^−16^), which was located downstream of *PABPN1*. A conditional analysis identified four independently associated SNPs at this locus, spanning a 9-Mb window that consisted of several meiosis-related genes, including *REC8*, *REC114*, and *FMN1* (Table 1). The *REC8* gene has been previously reported to associate with recombination rate in cattle [29]. The top associated SNP at the second locus on chromosome 10 was rs43640523 (*P*_female_ = 8.96×10^−23^; *P*_male_ = 9.10×10^−13^). This SNP was located 10 kb downstream of *NEK9* that was related to spindle organization and cell cycle progression during mouse oocyte formation [40]. A conditional analysis adjusting for the top SNP in this locus indicated a single underlying QTL in this region.

**Table 1:** Independent SNPs associated with genome-wide recombination rate in females and males.

SNP_rs	SNP	Ch r	Position^a^	Single-Marker Analysis						Joint Analysis				Gene^b^	Anotation
				Female			Male			Female		Male			
				Freq	Beta	*P*-val	Freq	Beta	*P*-val	Beta	*P*-val	Beta	*P*-val		
rs110661033	ARS-BFGL-NGS-83544	1	158140250	0.09	0.52	4.1×10^−26^	0.09	0.34	0.013	0.52	1.7×10^−30^	0.36	0.004	*PRDM9*	Downstream
rs109665521	BovineHD0300015148	3	49711874	0.23	-0.39	1.5×10^−13^	0.23	-0.52	6.7×10^−05^	-0.40	3.7×10^−17^	-0.53	6.4×10^−06^	*GCLM*	Upstream
rs136642773	BovineHD0300020445	3	69231601	0.75	0.27	1.0×10^−10^	0.74	0.10	0.375	0.28	5.3×10^−14^	0.10	0.282	*MSH4*	Intron
rs110253089	ARS-BFGL-NGS-117763	6	109108015	0.28	0.55	3.0×10^−51^	0.28	1.09	7.3×10^−30^	0.57	6.2×10^−64^	1.07	3.7×10^−33^	*CPLX1*	Intron
rs135733704	BovineHD1000006594	10	20216522	0.98	0.42	6.2×10^−06^	0.98	0.94	0.0003	0.74	5.3×10^−17^	1.44	3×10^−09^	*REC114*	Downstream
rs137264867	BovineHD1000006965	10	21437447	0.78	-0.59	2.6×10^−51^	0.77	-0.88	1.1×10^−16^	-0.64	3.2×10^−65^	-0.92	2.3×10^−20^	*PABPN1*	Downstream
rs135909314	BovineHD1000007192	10	22169580	0.88	-0.31	2.9×10^−11^	0.89	-0.37	0.005	-0.22	4.4×10^−07^	-0.14	0.251	/	mRNA
rs43623547	BovineHD1000009771	10	29619086	0.02	-0.56	1.7×10^−08^	0.02	-0.77	0.015	-0.54	7.7×10^−09^	-0.78	0.007	*FMN1*	Upstream
rs43640523	BTA-78285-no-rs	10	86717378	0.54	-0.32	9.0×10^−23^	0.54	-0.64	9.1×10^−13^	-0.34	5.3×10^−30^	-0.59	4.5×10^−13^	*NEK9*	Upstream
rs109452965	ARS-BFGL-NGS-84575	26	14912765	0.38	0.27	8.6×10^−17^	0.39	-0.06	0.477	0.26	4.4×10^−18^	-0.04	0.663	*CEP55*	Downstream
rs133252805	BovineHD2600008380	26	31387126	0.45	0.17	1.3×10^−07^	0.47	0.001	0.989	0.16	3.8×10^−08^	-0.001	0.986	*SMC3*	Upstream
rs137337293	BovineHD3000046354	X	34600788	0.94	0.02	0.845	0.95	0.72	1.3×10^−07^	-0.005	0.944	0.72	4.6×10^−09^	/	Intergenic
rs42382307	BTB-01224376	X	122124527	0.14	0.33	2.8×10^−08^	0.19	-0.19	0.059	0.32	1.1×10^−09^	-0.19	0.035	/	Intergenic

^a^ USDA-AGIL SNP coordinates

^b^ Bovine UMD 3.1 genome assembly

Although the top three associated loci were shared between sexes, differences between sexes were observed among the less significant associations. We observed a trend of smaller *P*-values in females in general (Table 1), indicating a difference in statistical power between the two sexes. In total, we identified six loci associated only in females and one locus associated only in males (Table 1 and Fig 5). The female-biased association on chromosome 1 peaked at rs110661033 (*P*_female_ = 4.14×10^−26^). This SNP was also nominally associated in males (*P*_male_ = 0.013), exhibiting the same direction of effect in both sexes. Taking into account the difference in power, this association is more likely to be shared between sexes than a female-specific effect. Moreover, this association was 35 kb downstream of the *PRDM9* gene, which has been associated with both recombination rate and hotspot usage in the two sexes [9,11,12]. Similarly, the association at rs109665521 (*P*_female_ = 1.53×10^−13^; *P*_male_ = 6.68×10^−5^) in the first locus on chromosome 3, showing the same effect direction in both sexes, are less likely to be female-specific.

Potential sex-specific associations with recombination rate were found at five loci on chromosomes 3, 26 and X, with one male-specific and four female-specific (Table 1). SNP rs137337293 on the X chromosome was associated only in the male (*P*_male_ = 1.27×10^−7^). Among the four female-specific associations, two were inside or near genes closely related to the meiotic pathway. Rs136642773 (*P*_female_ = 1.04×10^−10^) was located in the intron of *MSH4*, which is a meiosis-specific *MutS* homologue that affects crossing over [41,42]. SNP rs133252805 (*P*_female_ = 1.25×10^−7^) was upstream of *SMC3* that encodes a protein related to meiotic chromosomes and synaptonemal complexes [43]. The other two female-specific associations were observed at rs109452965 near *CEP55* (*P*_female_ = 8.6×10^−17^) and rs42382307 on the X chromosome (*P*_female_ = 2.8×10^−8^).

To comprehensively evaluate the associations and estimate their effects, we conducted a joint analysis by including all the significantly associated SNPs in one model as fixed effects (Table 1). As expected, the associated *P*-values become smaller from the joint analysis than in the single-marker analysis, for those independent associations in both males and females because of the reduced residual errors [44]. The largest difference/ratio between the *P*-values of the single-marker and joint analyses was 10^14^ for the association at rs137264867 in females.

### Genetic control of recombination hotspot usage in males and females

Based on the 1,792 male and 1,885 female recombination hotspots, we calculated the proportion of recombination events occurring in the hotspots genome-wide, i.e., hotspot usage, for the sire and dam in a three-generation family. To increase accuracy, we only included the high-quality meioses, where the offspring, the parent and the grandsire were genotyped by the 50K SNP chip. We also used the average of multiple measurements of hotspot usage as the phenotype, resulting in a sample size of 1,772 and 12,756 for males and females respectively. We then tested the association between hotspot usage and each of the 310,442 imputed SNPs. To evaluate the effect of different definitions of hotspot, we tested a range of cutoff values, 2, 2.5, 3, 5, and 10 standard deviations, and found that the cutoff value of 2.5 standard deviations had the clearest signal for the association between *PRDM9* and hotspot usage.

**Fig 6:**
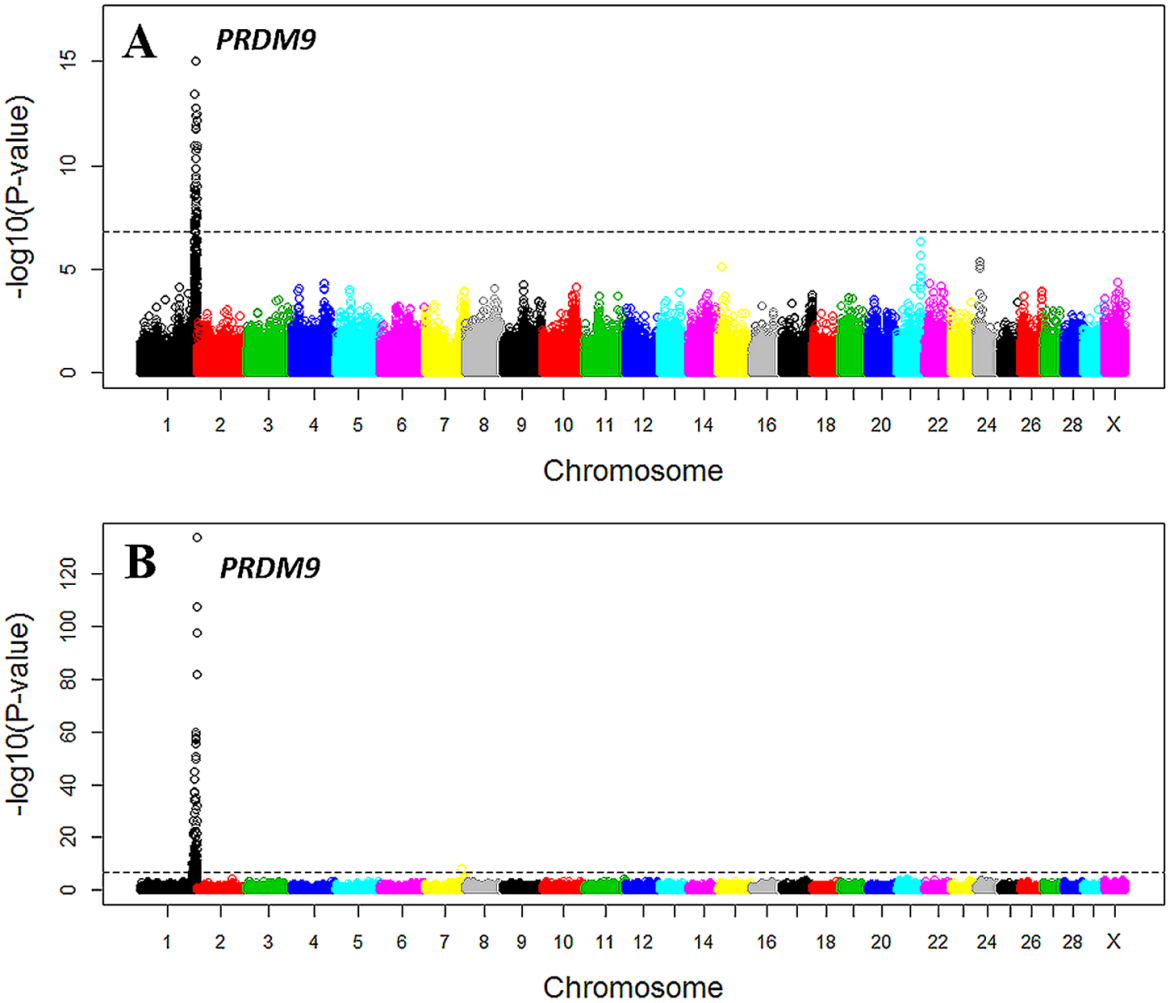
Manhattan plot of the GWAS of genome-wide hotspot usage for males (A) and females (B). Different colors were used to distinguish the 29 chromosomes. The genome-wide significance level of 1.6×10^−7^ was shown by the horizontal dotted line. USDA-AGIL SNP coordinates were used for plotting, which placed *PRDM9*-linked SNPs to the end of Chromosome 1. Note that UMD3.1 assembly placed *PRDM9* near the middle of Chromosome 1.

The GWAS results indicated that recombination hotspot usage was much less polygenic than recombination rate, because we identified a single associated locus in both males and females for hotspot usage (Table 2 and Fig 6 and S6 Fig) and thirteen associated loci for recombination rate (Table 1). The top SNP was rs110661033 located 35 kb downstream of *PRDM9* (*P*_female_ = 2.20×10^−134^; *P*_male_ = 6.59×10^−13^). This SNP was also associated with genome-wide recombination rate (Table 1). Animals that carry one copy of the minor allele (G) of this SNP (MAF = 0.09), on average, showed a decrease of 2% and 1% in hotspot usage in females and males respectively (Table 2). However, the effect of the association with recombination rate was just the opposite: one copy of the major allele (A) had a decrease of 0.52 and 0.34 crossovers in female and male recombination rates respectively. By adjusting for the effect of rs110661033, the conditional analysis identified a second, independent association at rs132965246 in males only (*P*_male_ = 1.03×10^−15^). In females, this SNP was only nominally associated with hotspot usage from the joint analysis, but with an opposite effect (Table 2).

**Table 2:** Independent SNPs associated with hotspot usage in females and males.

SNP_rs	SNP	Ch r	Position^a^	Single-Marker Analysis						Joint Analysis				Gene^b^	Anotation
				Female			Male			Female		Male			
				Freq	Beta	*P*-val	Freq	Beta	*P*-val	Beta	*P*-val	Beta	*P*-val		
rs132965246	BovineHD0100045523	1	155810889	0.82	-0.0003	0.704	0.83	-0.009	1.0×10^−15^	0.002	0.008	-0.008	2.1×10^−15^	*/*	/
rs110661033	ARS-BFGL-NGS-83544	1	158140250	0.09	-0.02	2.2×10^−134^	0.09	-0.01	6.6×10^−13^	-0.02	1.2×10^−142^	-0.01	1.8×10^−12^	*PRDM9*	Downstream

^a^ USDA-AGIL SNP coordinates

^b^ Bovine UMD 3.1 genome assembly

Note that the locations of several associated SNPs near *PRDM9* were different between the UMD3.1 assembly and the USDA-AGIL SNP coordinates: UMD 3.1 assembly placed *PRDM9* near the middle of chromosome 1 while AGIL map moved these *PRDM9*-linked SNPs to the end of the chromosome (Fig 6). The Baylor Btau_4.6.1 genome assembly also placed the *PRDM9* gene to the end of chromosome 1 [45]. To further validate the location of *PRDM9*, we investigated the pairwise linkage disequilibrium patterns between the top associated SNP, rs110661033, and all other SNPs on chromosome 1. The results supported that the *PRDM9* associated with cattle recombination is located to the end of chromosome 1, because rs110661033 had strongest LD with SNPs at the end of the chromosome and lower LD with SNPs away from this mapped location (S7 Fig).

### Trends in male and female recombination over time

After adjustment for the effects of SNP array and inbreeding in both sexes and for number of offspring in bulls, we identified a decreasing trend in genome-wide recombination rate in males in the past decades (S8 Fig). As described in previous sections, we estimated the genome-wide recombination rate and hotspot usage for bulls that were born between the years 1970 and 2012 and for cows born between 1990 and 2012, allowing us to evaluate the trends of these recombination features over the years. Male recombination rate dropped from 27.1 to 24.7 Morgans from 1974 to 1990 and then continued the decrease but with a slower speed after 1990. The decreasing trend in females is not as clear as that in males (S8 Fig). This declining recombination rate with artificial selection is consistent with the recent empirical evidence that domestic animals exhibit lower recombination rate than their wild counterparts [46]. In addition, the decreasing trend in recombination rate partially explained the shorter recombination maps compared with existing maps because a major proportion of the cattle used in this study were born after 2000. However, recombination hotspot usage showed a non-significant, but two-stage trend in both sexes: an increasing trend before 2006 and a reduction after 2006 (S9 Fig).

## Discussion

The next decade is predicted to witness a substantial growth in global population and a possibly larger increase in demand for animal products due to spreading affluence. To meet the growing demand for meat and dairy products, cattle industries have begun to adopt alternative strategies for increasing production through genomic selection [47,48]. Understanding of the genomic features of cattle, including the mechanisms of meiotic recombination, genetic loci that are associated with recombination, and the high-resolution recombination maps, is directly relevant for genomic evaluation [49,50]. Based on SNP genotypes from over half a million Holstein cattle with pedigree information, the present study reported recombination maps for both males and females, identified recombination hotspots in each sex, provided in-depth insights into the genetic basis of individual differences in recombination, and demonstrated a decreasing trend over time in recombination rate that coincided with a period of steady selection response to artificial selection for milk production.

This study reported cattle-specific features of recombination as well as features that are shared between cattle and other mammals. We provided compelling evidence of sex differences in recombination rate in cattle, which is consistent with the results that were previously reported for most of the mammalian species [9,18,51]. However, in striking contrast to humans and mice where the male recombination map is shorter, our results demonstrated that the male recombination map of cattle was over 10% longer than that of the females. We also showed that the higher recombination rate in males was most pronounced near the telomeres. Interestingly, higher male recombination rates in the subtelomeric regions have been consistently reported in humans and mice, despite the differences between cattle and other mammals in the overall patterns of sex-biased recombination rate [9,19,37,38]. In addition, a decrease in recombination rate at the centers of acrocentric chromosomes in cattle possibly due to crossover interference was also observed in humans (S10 Fig) and mice [38]. A further comparison between our GWAS results and two QTL mapping studies in mouse revealed a common QTL region encompassing *MSH4* that was orthologous between cattle and mouse [52,53].

Although the biological significance for a longer male map in contrast to most mammalian species is unclear, we speculate that cattle domestication, which was estimated to have begun approximately 10,000 to 11,000 years ago [54], and the intense artificial selection targeting specific traits thereafter, could be a plausible explanation. In the past, the breeding practices in dairy cattle have put more selection pressure on bulls than on cows. Based on several theories of recombination rate evolution, this male-biased selection may lead to a higher recombination rate in bulls if selection has a direct or indirect, positive effect on recombination [55–57]. Such a pattern of a longer male map was also observed in sheep [58], which is presumed to have been domesticated during the same contemporary period as dairy was domesticated and then underwent similar male-biased selective breeding [59]. In contrast to domestic sheep, the female recombination rate of the wild bighorn sheep was reported to be 12% greater than that of the male [60].

Based on 59,309 autosomal SNPs, we constructed cattle male and female recombination maps, 25.5 and 23.2 Morgans in length. A previous study that exclusively used bulls also reported a similar length of the male map [29]. Compared to the previously documented cattle linkage maps that were based on a small number of markers with limited sample sizes [24,27,28,61], our sex-specific maps were shorter in length. Such discrepancy could be due to several factors. Errors in the physical map or in the genotypes can inflate the number of identified recombination crossovers and increase the length of the genetic map [62]. Previously documented linkage maps were based on a smaller number of RFLP or microsatellite markers, which could potentially bias the estimates. Our simulation studies further validated the power of identifying crossovers in this study (97.6%) and the accuracy of our estimates of the length of recombination maps. Moreover, the previous studies with a smaller number of markers were probably less powerful, potentially contributing to this difference.

We found a significantly decreasing trend in recombination rate in males from the analyses of recombination in the past forty years. Such decline in recombination rate in the past forty years coincided with the steady increase in milk production and decrease in fertility, a result of the intensive artificial selection in cattle breeding [47,63,64]. Although recombination generally increases selection efficiency by providing more combinations of genetic alleles [3], recombination likely was selected against in cattle breeding that predominantly occurred in males. In cattle breeding, bulls tended to carry more desired chromosomes so that a male progeny that inherited the most chromosome segments from an elite sire would have better performance and more chance to be selected for breeding. In other words, the cattle breeding favored paternal haplotypes that were not or less mixed with the maternal haplotypes during meiosis. Therefore, a sex-biased cattle breeding and selection could potentially decrease the number of recombination in a short period and likely explain the reduction of recombination rate in cattle, particularly in males. To evaluate whether the decrease in recombination rate is correlated with systematic changes in allele frequencies of associated genetic variants, we calculated the frequencies of the alleles that increase recombination rate for associated SNPs over years but found no clear patterns (S11 Fig). Inbreeding decreases the power of identifying crossovers through reducing the number of heterozygote SNPs per individual, so we adjusted for the effect of inbreeding by including the genomic inbreeding coefficient of the individual and the numbers of informative (phased heterozygote) SNPs in both the parent and offspring in a linear model. As expected, we found a negative association between inbreeding coefficient and number of recombination events in both sexes.

Our GWAS analyses identified several loci influencing genome-wide recombination rate. Some of these loci had significant influence in both sexes (*PRDM9*, *GCLM*, *CPLX1*, *PABP1*, *REC114*, *FMN1*, and *NEK9*), and some of them were potentially sex-specific (*MSH4*, *CEP55* and *SMC3*). We also confirmed the putative role of *PRDM9* in the genome-wide recombination rate in both sexes. From GWAS of hotspot usage, we confirmed the cattle *PRDM9* gene in both sexes to be the paralogue on chromosome 1 in our population, although the cattle genome encompasses multiple paralogues of *PRDM9* and a previous study localized the associated *PRDM9* to chromosome X [29].

To better understand the sex difference in recombination rate in the subtelomeric regions, we conducted additional GWAS of subtelomeric recombination rates, in which the phenotype was the number of crossovers that occurred in the last 15% of each chromosome. Compared with the GWAS of genome-wide recombination rate, the subtelomere GWAS identified a smaller number of associations that have already been found from the GWAS of genome-wide recombination rate, including the loci on chromosomes 1, 6, 10, and 26 (S4 Table). While many of these associations showed a larger effect in males than in females, the association at *PRDM9* exhibited the same effect size in the two sexes, suggesting a possible unique role of *PRDM9* in the subtelomeric recombination. Interestingly, the effect size of the *PRDM9* association with genome-wide and subtelomeric recombination rates was the same in males, which might be related to the large number of male recombination hotspots in subtelomeric regions.

Recombination rate is positively correlated with physical distances between SNPs. In this study, we used the original recombination rate between two SNPs without adjusting for physical distance to define recombination hotspots for several reasons. First, the SNPs on genotyping chips were about evenly distributed. Second, our hotspot definition was supported by the identification of association between hotspot usage and *PRDM9*, consistent with results in human and mouse. We tested a range of cutoff values to define hotspots from 2 to 10 standard deviations and the association between *PRDM9* and hotspot usage was consistently identified. Third, the original recombination rate without adjustment for physical distance is unaffected by inaccurate physical distances in the genome assembly. To evaluate if our results were biased by the physical distances between SNPs, we standardized recombination rate by physical distance. With this correction, we noticed several spurious hotspots that had a very small physical length but a moderate recombination rate, suggesting the existence of potentially inaccurate physical distances. To eliminate biased correction for physical distance due to potentially inaccurate physical distances, we filtered all SNP intervals shorter than 500 bp and calculated a standardized recombination rate between SNP pairs by dividing the original recombination rate by its physical distance. With this correction, the pattern of recombination rate along the chromosome (S12 Fig) was similar to that without this correction (Fig 4). The standardized recombination rates between SNP intervals were less variable so we used a cutoff of 0.6 standard deviations to be able to identify 2,875 and 3,005 male and female recombination hotspots, respectively. Using these hotspots with the correction for physical distance, the GWAS of hotspot usage identified the association at *PRDM9* in females (S13 Fig), but with larger *P*-values (less significant) than those from the original GWAS (Fig 6). In males, the association at *PRDM9* was only nominally significant (S13 Fig). Taken together, we recommend using the original recombination rate for hotspot definition without adjustment for physical distances, and the quality of our results is evidenced by the shorter recombination maps and the confirmation of several known recombination genes including *PRDM9* and *CPLX1*.

In conclusion, our large-sample study reveals new insights into the cattle meiotic recombination and its genetic basis by offering male and female recombination maps, a sex difference in recombination rate that predominantly occurred in subtelomeres, and genomic loci associated with recombination rate and hotspot usage in the two sexes. Our study clearly delineates that the genomic resources accumulated during many years of genetic evaluation in cattle provide valuable opportunities for understanding cattle genetics including genome recombination.

## Materials and Methods

### Three-generation families from the large pedigree

To infer recombination events and compare sex differences, we extracted a total of 185,917 three-generation families from a large pedigree of Holstein cattle maintained in the Animal Genomics and Improvement Laboratory (AGIL) at USDA, with one offspring, both parents and two grandsires with SNP genotypes in each family (Fig 1). In each of the 185,917 families, we inferred recombination events for a paternal meiosis from the sire/offspring pair and a maternal meiosis from the dam/offspring pair. For recombination map construction and GWAS of hotspot usage, we only included the highest-quality or most informative meioses where the offspring, the parent, and the grandsire were genotyped by 50K SNP chips, resulting in a total of 70,715 male and 61,616 female meioses. For GWAS analysis of genome-wide recombination rate, we included all paternal and maternal meioses from the 185,917 families regardless of the number of SNPs genotyped.

The animals in the selected families were genotyped by various genotyping assays (S1 Table), ranging from 3K to 770K SNPs [33]. The Illumina BovineSNP50 v1 chip with 56,947 SNPs, v2 chip with 54,609 SNPs, the high-density (HD) chip with 777,962 SNPs, and the GeneSeek HD chip with 77,068 SNPs are referred to as the 50K chip, as we used a combined set of >50K SNPs [65]. The Zoetis BovineLD chip with 10,555 SNPs, the GeneSeek Genomic Profiler v1 and v2 chips with 8,042 and 8,415 SNPs, the Illumina BovineLD BeadChip with 6,785 SNPs, the Illumina Bovine3K BeadChip with 2,708 SNPs are referred to herein as 10K, 8K, 7K, and 3K, respectively. The chips were designed as mostly nested with the higher-density chip including SNPs on the lower-density ones. Although the offspring and two parents were genotyped by various SNP chips, the grandsires were mostly genotyped by the 50K SNP chips. Note that the granddam was not necessarily genotyped in the selected three-generation families. Depending on the number of genotyped granddams, we collected 67,690, 76,318, and 41,909 families with two, one and zero genotyped granddams, respectively. Note that an animal may appear in more than one family based on the pedigree structure, especially for bulls that have hundreds of progeny.

### Haplotype phasing and recombination identification

To study recombination, we included up to 59,309 genome-wide SNPs after quality control filtering and used the USDA-AGIL SNP coordinates that showed a higher quality than the UMD3.1 assembly [30,33]. Previously, several SNPs were relocated from the UMD3.1 assembly to the USDA-AIGL coordinates in cooperation with researchers from the University of Missouri (R. D. Schnabel), and the University of Guelph (M. Sargolzaei and J. Johnston). From pairwise linkage analysis in this study, we also removed nine suspicious SNPs that exhibited suspicious linkage disequilibrium (LD) patterns with SNPs on the same chromosome (S2 Fig and S3 Table). Due to the low quality of the genome assembly, we excluded the X chromosome from recombination calculation in this study.

To compare sex differences on an equal footing, we phased the raw genotypes for the paternal and maternal meioses within a three-generation family without using the possible additional information from multiple offspring [66], because bulls generally had many more progeny than cows. In each of the three-generation families, we inferred the paternal and maternal haplotypes of the offspring based on the genotypes of the two parents, and also inferred the paternal and maternal haplotypes for both parents based on the genotypes of the grandparents [29,66]. Homozygous genotypes were phased trivially and the heterozygous genotypes were phased whenever the genotypes of the two parents are not heterozygous simultaneously. The parent-of-origin was then assigned to each allele after phasing to determine paternal and maternal haplotypes.

After phasing the genotypes of the offspring and parents, we inferred recombination events in the paternal and maternal haplotypes of the offspring by comparing the offspring’s paternal haplotype to the two haplotypes of the sire as well as by comparing the offspring’s maternal haplotype to the two haplotypes of the dam. In the offspring haplotype, a recombination event was defined as a transition from the parent's paternal to maternal haplotype or vice versa. Note that the recombination events defined here were observed crossovers so that the number of observed crossovers could differ from the number of true crossovers when multiple crossover events occurred at the same site. This potential inconsistency between observed and true crossovers typically is addressed by a map function that translates a recombination frequency into a map distance in terms of crossovers. However, the physical distances between two adjacent SNPs were small so that the use of a map function virtually would not make a numerical difference. Therefore, our estimates of crossovers based on recombinants should be close to the true number of underlying crossovers. To further reduce false positives, we required a crossover call to be supported by at least three consecutive informative heterozygous SNPs [9]. In total, we identified ~4.5 million paternal and ~4.0 million maternal recombination events from the total of 185,917 paternal and maternal meioses, respectively. To ensure that our results do not depend strongly on the cutoffs, we repeated the analysis by using a different cutoff value of 5 consecutive informative markers and the number of identified crossovers was only reduced by 0.2%.

### Construction of recombination maps using an EM algorithm

A recombination event was assigned to a region spanned by two informative SNPs that may not be adjacent to each other. To construct a recombination map, we used an EM-algorithm to calculate the probability of crossing over per meiosis or recombination rate between each pair of consecutive SNPs based on the observed crossover regions [9]. After an initiation step to assign an expected count of 1/*m* to each of the *m* adjacent SNP intervals in a crossover region, the EM algorithm proceeded in the following iteration steps: 1) M-step: considering a total of *n* meioses, the overall expected count attributed to a SNP interval divided by *n* were the maximum likelihood estimate of the probability, and 2) E-step: for a crossover region, the expected count assigned to a SNP interval was estimated as proportional to the current estimate of the probability of crossover for that SNP interval. The M and E steps were iterated until convergence.

We constructed the male and female recombination maps for 59,309 autosomal SNPs based on >1.8 million paternal and >1.4 million maternal recombination crossovers, which were identified from 70,715 male and 61,616 female meioses that are most informative where the offspring, the parent, and the grandsire were genotyped by 50K SNP chips. Note that some granddams were genotyped by a low density chip or even not genotyped. As described earlier, a correction for the number of SNPs of the granddam was employed in the two sexes separately such that the total number of crossovers after correction was the same regardless of the SNP numbers for granddams. We used the expected number of crossovers per meiosis or recombination rate between adjacent SNPs as the genetic distance in our recombination map, since one crossover event on average corresponds to a genetic distance of 1 Morgan and recombination rate is almost the same as genetic distance for small intervals [9,67]. Alternatively, using Haldane’s map function with crossover interference [68], the male and female maps were slightly longer, 25.6 and 23.3 Morgans in length respectively.

### Identification of recombination hotspots with validation from pairwise linkage disequilibrium analysis

We defined recombination hotspots as the SNP intervals with a recombination rate >2.5 standard deviations from the genome-wide average in males and females separately, because 2.5 standard deviations are highly significant departures from the average recombination in cattle given our large sample sizes in both sexes, consistent with the observations from a recent recombination study in cattle [29]. We tested a range of cutoff values, 2, 2.5, 3, 5, and 10 standard deviations, and found that the cutoff value of 2.5 standard deviations showed the clearest signal for the association between *PRDM9* and recombination hotspot usage. For validation purposes, we also defined recombination hotspots via using a standardized recombination rate that was calculated by dividing the original recombination rate between two SNPs by the physical length. After this adjustment of physical lengths, the standard recombination rates vary even less so we used a cutoff value of 0.6 standard deviations to define recombination hotspots.

To ensure the quality of the hotspots identified, we calculated pairwise linkage disequilibrium (LD) statistics between each of the SNPs in or near a recombination hotspot and all other SNPs on the same chromosome using Locusmap [35]. The pairwise LD was evaluated by a LOD score and an estimated recombination rate. For a SNP with correct physical position, the LOD scores should peak near the SNP and decrease when moving away in the two directions. The recombination rate should follow the opposite pattern with small values near the SNP and increasing with the distance. Any obvious deviations from these expected LD patterns suggest a possible error in the SNP coordinate and thus a false recombination hotspot (S2 Fig). Nine suspicious SNPs exhibiting unexpected LD patterns together with seven originally identified recombination hotspots were removed (S5 Table).

### Number of SNPs and power of recombination identification

As expected, the power for identifying recombination events was affected by the number of genotyped SNPs. For male meiosis, the average number of crossovers identified varied from 25.9 to 17.7 depending on the number of measured SNPs of the animals in a three-generation family (S2 Table). Overall, more crossovers were identified when the number of SNPs increased, except for a few categories that had a small sample size and thus large noise. A similar pattern was also observed for female meioses, where the number of crossovers ranged between 23.9 and 16.0 (S3 Table To account for this effect of SNP numbers, we used the highest-quality meioses where all animals were genotyped by 50K SNP chip as a reference to correct the number of crossovers identified in other meioses. After correction, the average number of crossovers was equal to 25.5 in males and 23.2 in females.

To evaluate the power for identifying crossovers using 50K SNP genotypes, we simulated 50K SNP genotypes for all animals in the real pedigree and used the same phasing and crossover identification procedures as described in previous sections. By defining a positive result as an identified crossover interval overlapping with the true location of a crossover, we calculated the power of crossover identification as the proportion of positive results across all simulated crossover events. As a result, our approach had a power of 97.6% for the three-generation families genotyped by 50K SNP chip, which means that on average only 2.4% crossovers were missed in our recombination map.

### Smooth spline plot of recombination rate with chromosomal locations and time

To evaluate the relationship between recombination features and potentially related factors, we fitted a smooth spline model of the male and female recombination rates on relative chromosomal locations or time using the smooth.spline function implemented in R 3.1.1 [69]. We calculated a relative physical position for each of the SNP intervals by dividing the original physical position by the corresponding chromosome length. For the analysis of chromosome location effect on recombination, we used those meioses for which all the required individuals were genotyped by 50K SNP chip and removed 2-Mb regions to the end of all chromosomes where the power of identifying crossovers is low [9]. For the analysis of time trend, we used all the meioses and adjusted for the effects of SNP chips and inbreeding, as well as effect of influential bulls by accounting for the number of progeny for bulls. Specifically, the correction was conducted with a linear model for the number of crossovers by fitting fixed effects for the categories of SNP chips of the offspring, parent and two grandparents, genomic inbreeding coefficient of the parent, the number of phased heterozygous SNPs of the offspring and parent, quadratic and cubic terms of the two numbers of informative SNPs, and the interaction terms between them. As expected, we found a negative association between inbreeding coefficient and number of recombination events in both sexes (Males: *β* = −0.28 and *P*-value = 0.006; Females: *β* = −0.11 and *P*-value = 3.2 × 10^−12^). To investigate the common pattern across the 29 autosomes, we pooled all the autosomes together using the relative physical position and fitted a smooth spline model for all the data combined (Fig 4). We also fitted a smooth spline model for each of the chromosomes individually (S3 Fig). A degree of freedom of five was used in all the smooth spline modeling. A similar smooth spline model was fitted for the analysis of time trend of recombination rate and hotspot usage (S8, S9, S10 and S12 Figs).

### GWAS of genome-wide recombination rate and hotspot usage using a linear mixed model with variable residual variances

We estimated the number of recombination events for maternal and paternal meioses in each of the 185,917 three-generation families, which were then assigned to the sire and dam in the family. Each sire or dam may have multiple phenotypic measurements when appearing in more than one family, and we calculated the average of the multiple measurements as the phenotype for genome-wide recombination rate. A total of 3,224 bulls and 53,125 cows were included in the GWAS of genome-wide recombination rate. We corrected the originally estimated number of recombination events by the number of measured SNPs of the animals for each meiosis so that the average number of crossovers was the same regardless of the genotyping assays (S2 and S3 Tables).

We calculated the genome-wide proportions of crossovers occurring in the hotspots, i.e. hotspot usage, for the 70,715 male and 61,616 female meioses that are most informative where the offspring, the parent and the grandsire were genotyped by 50K SNP chips. After assigning the estimated hotspot usage to the sire or dam for each meiosis, we used the average of the multiple measurements as the phenotype, resulting in a sample size of 1,772 and 12,756 in males and females, respectively.

Imputed genotypes of 777,962 SNPs on the Illumina BovineHD Genotyping BeadChip (HD) were obtained by running Findhap on measured genotypes with 3K to 50K SNP chips using a reference population of 2,433 animals directly measured with HD SNP chips [33,70]. After filtering SNPs exhibiting redundancy, very high linkage disequilibrium (*r* > 0.95), or small minor allele frequency (MAF < 0.001), we retained over 310K genome-wide SNPs in the association studies [33].

We tested for association between each SNP and a phenotype using a linear mixed model with variable residual variances that are inversely proportional to the number of repeated measures of the phenotype, i.e., residual variance is smaller for individuals with more measurements of the phenotype. The model equation in matrix notation is

y=Xg+Za+e

where **y** = a vector of the phenotype, **X** = a design matrix of the fixed effects **g**, including a population mean and the additive effect of the candidate SNP, **Z** = a design matrix for a random animal effect **a**, and **e** = a vector of random residuals. We assume that $a\;\sim \;N\left(0,A\sigma_{a}^{2}\right)$ and $e\;\sim \;N\left(0,R\sigma_{e}^{2}\right)$, where **A** is the genomic relationship matrix and **R** is a diagonal matrix with the *i*th diagonal element equal to 1/*w*, where *w* is the number of phenotypic measurements for the *i*th animal.

This model has been implemented in the MMAP software package with optimized computing [71,72], which can finish a GWAS analysis with 53,125 samples and 310K SNPs in hours using 32 CPU cores of the high-performance computer at USDA-AGIL. The model was empirically validated by observing no inflation in the quantile-quantile plots (QQ-plot) of the GWAS *P*-values for both recombination rate and hotspot usage in this study (S5 and S6 Figs).

## Supporting Information

S1 Fig Smooth spline plotting of density of informative SNPs in males and females along the chromosome. (DOCX)

S2 Fig Examples of normal, expected linkage disequilibrium patterns (A) and suspicious linkage disequilibrium patterns (B and C) between a SNP near a recombination hotspot and all other SNPs on the same chromosome. (DOCX)

S3 Fig Smooth spline plotting of recombination rate versus relative physical locations by autosomes. (DOCX)

S4 Fig Smooth spline plotting of recombination rate versus relative physical locations for single-crossover and double crossover meioses in the two sexes. (DOCX)

S5 Fig QQ-plot for the GWAS of recombination rate in males (A) and females (B). (DOCX)

S6 Fig QQ-plot for the GWAS of hotspot usage in males (A) and females (B). (DOCX)

S7 Fig Pairwise linkage disequilibrium patterns for LOD score (A) and recombination rate (B) between the top associated SNP, rs110661033 or ARS-BFGL-NGS-83544, near PRDM9 and all other SNPs on the same chromosome. (DOCX)

S8 Fig Time trend of recombination rate using a scatter plot and a smooth spline in males (A) and females (B), and a zoomed-in plot after 1990 in both sexes (C). (DOCX)

S9 Fig Time trend of hotspot usage using a scatter plot and a smooth spline in males (A) and females (B), and a zoomed-in plot after 1990 in both sexes (C). (DOCX)

S10 Fig Smooth spline plotting of recombination rate versus relative physical locations in humans for all autosomes (A) and for acrocentric chromosomes (B), including chr13, chr14, chr15, chr21 and chr22. (DOCX)

S11 Fig Frequency change over years for alleles with positive effect on recombination rate for 5 SNPs in Table 1. (DOCX)

S12 Fig Smooth spline plotting of recombination rate per 100 kb distance versus relative physical locations with correction for physical distance of each SNP interval. (DOCX)

S13 Fig Manhattan plots for the GWAS of hotspot usage with correction for physical distance of each SNP interval in males and females. (DOCX)

S1 Table SNP chips, the number of SNPs and the number of genotyped animals used in this study. (DOCX)

S2 Table The number of recombination events identified and SNP chips used for paternal meioses. (DOCX)

S3 Table The number of recombination events identified and SNP chips used for maternal meioses. (DOCX)

S4 Table SNPs associated with subtelomeric recombination rate in females and males. (DOCX)

S5 Table SNPs removed due to suspicious LD patterns near recombination hotspots. (DOCX)
