# Supplementary material for: Cattle Sex-Specific Recombination and Genetic Control from a Large Pedigree Analysis
Source: PLoS Genet. 2015 Nov 5;11(11):e1005387. doi: 10.1371/journal.pgen.1005387 (PMC4634960; doi:10.1371/journal.pgen.1005387)
Supplement: S1 Fig — (DOCX) [file pgen.1005387.s001.docx]

**Figure S1. Smooth spline plotting of density of informative SNPs in males and females along the chromosome.** There are no clear difference in the density of informative SNPs between males and females.

**
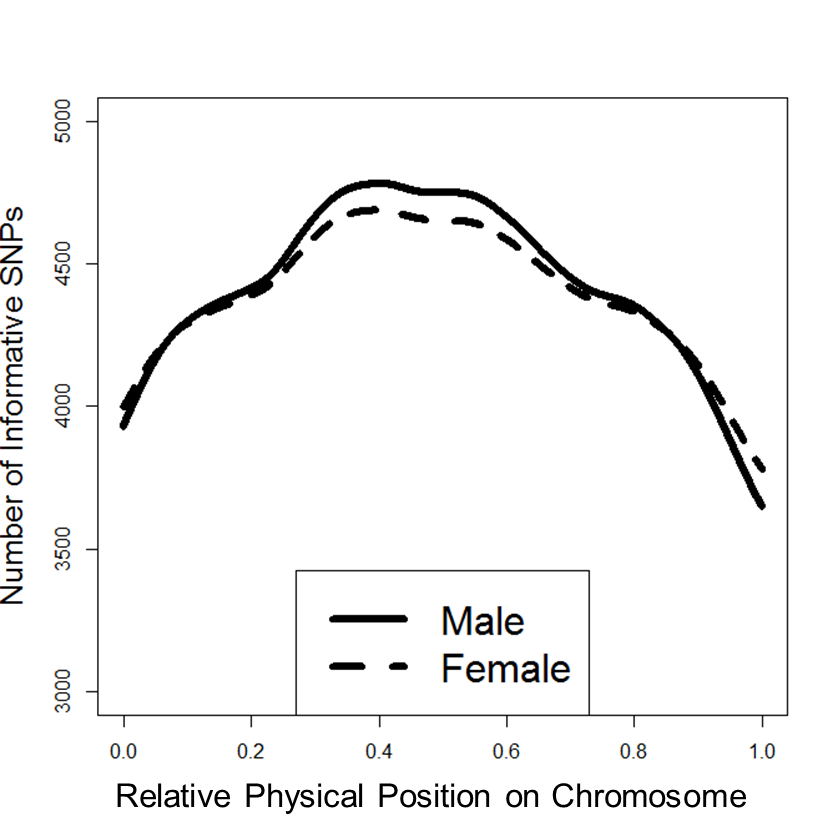
**
