# Supplementary material for: Cattle Sex-Specific Recombination and Genetic Control from a Large Pedigree Analysis
Source: PLoS Genet. 2015 Nov 5;11(11):e1005387. doi: 10.1371/journal.pgen.1005387 (PMC4634960; doi:10.1371/journal.pgen.1005387)
Supplement: S2 Fig — (DOCX) [file pgen.1005387.s002.docx]

**Figure S2. Examples of normal, expected linkage disequilibrium patterns (A) and suspicious linkage disequilibrium patterns (B and C) between a SNP near a recombination hotspot and all other SNPs on the same chromosome.** Vertical strait line denotes the location of the SNP being tested.


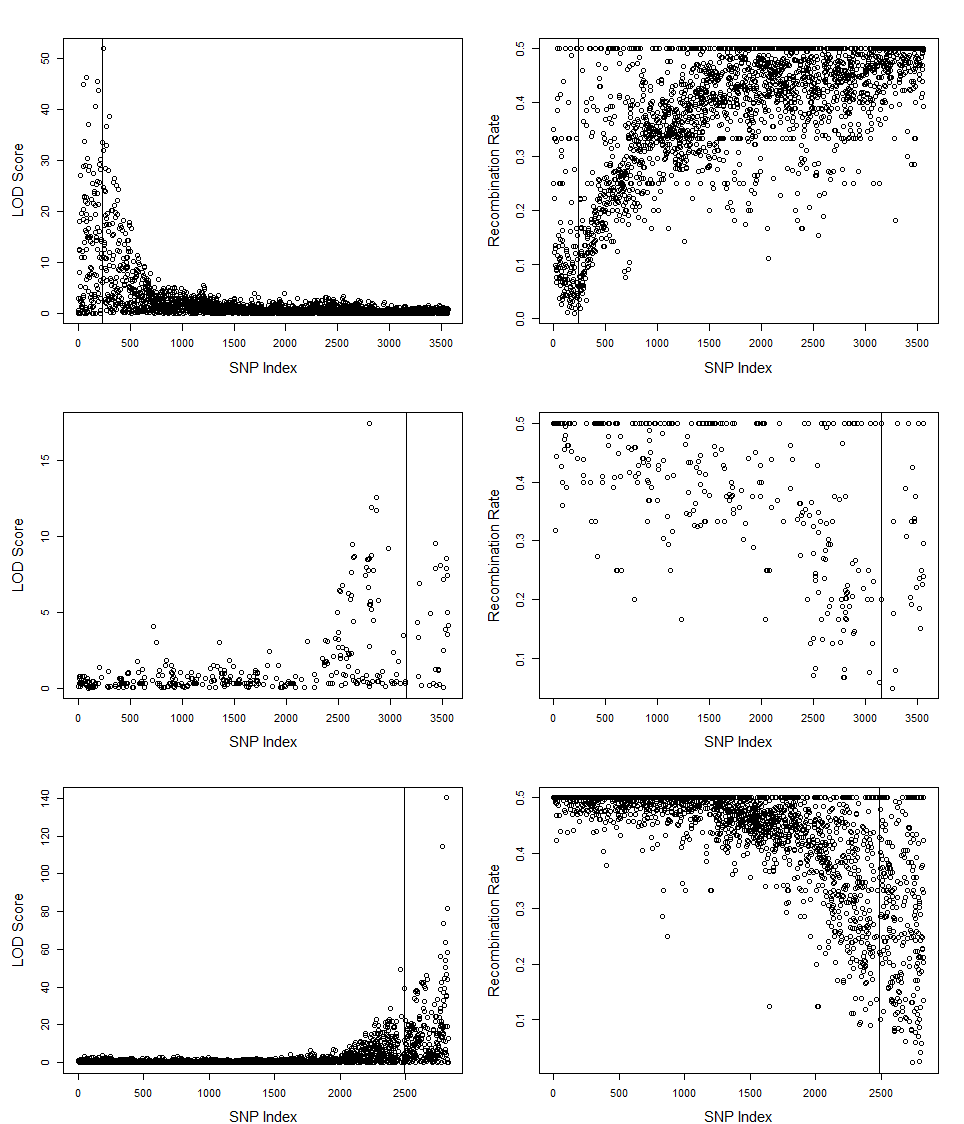


**C**

**B**

**A**
