# Supplementary material for: Cattle Sex-Specific Recombination and Genetic Control from a Large Pedigree Analysis
Source: PLoS Genet. 2015 Nov 5;11(11):e1005387. doi: 10.1371/journal.pgen.1005387 (PMC4634960; doi:10.1371/journal.pgen.1005387)

**Figure S3. Smooth spline plotting of recombination rate versus relative physical locations by autosomes.**


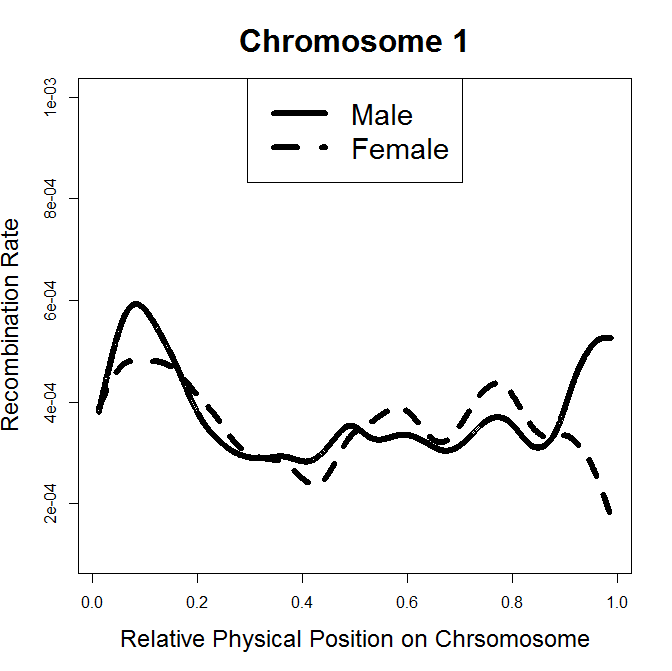

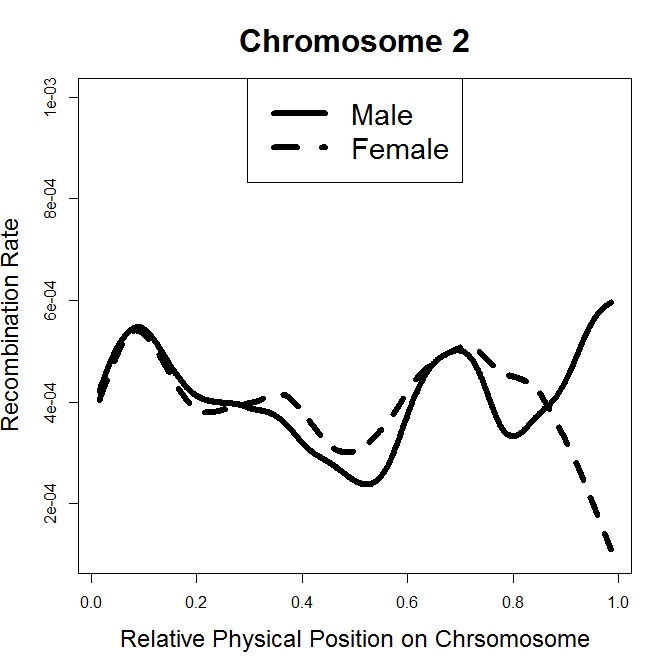

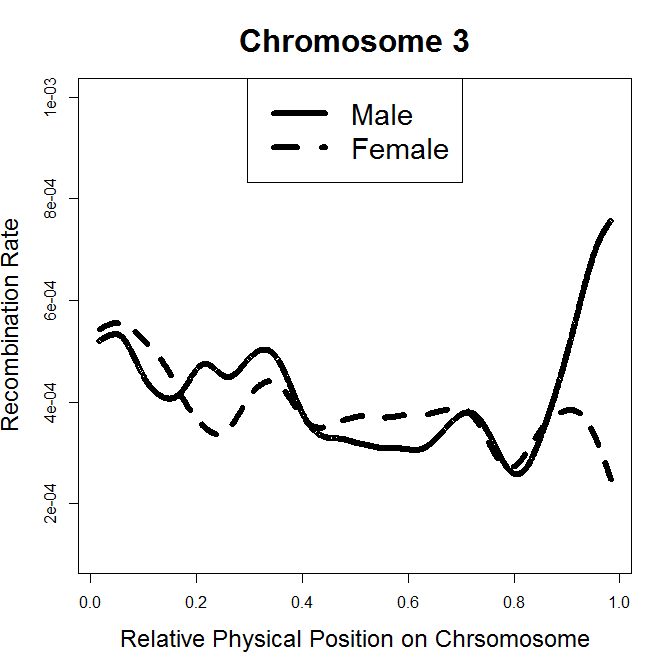


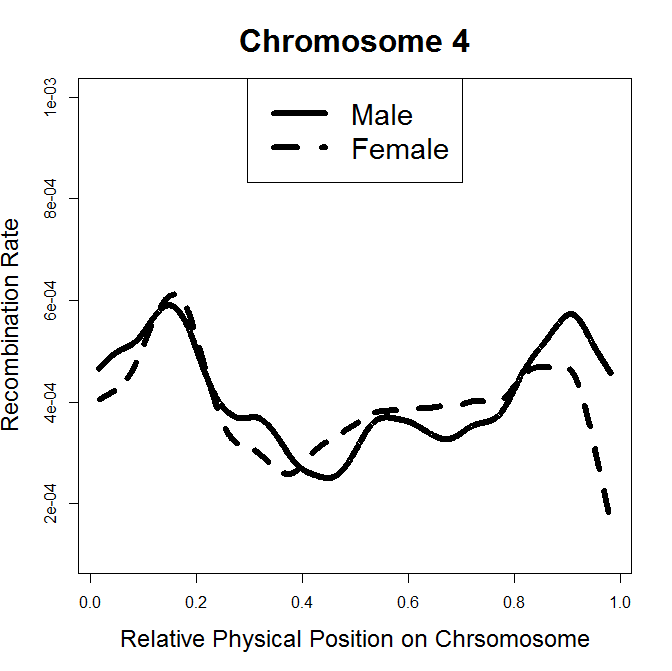

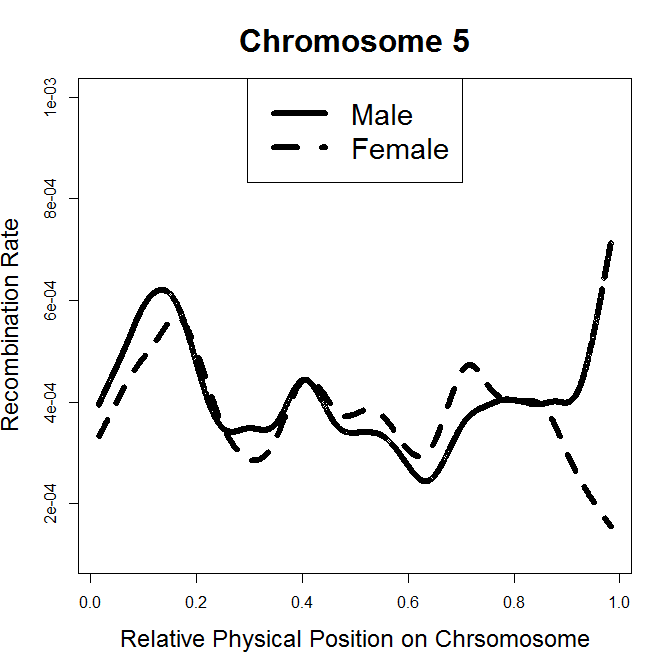

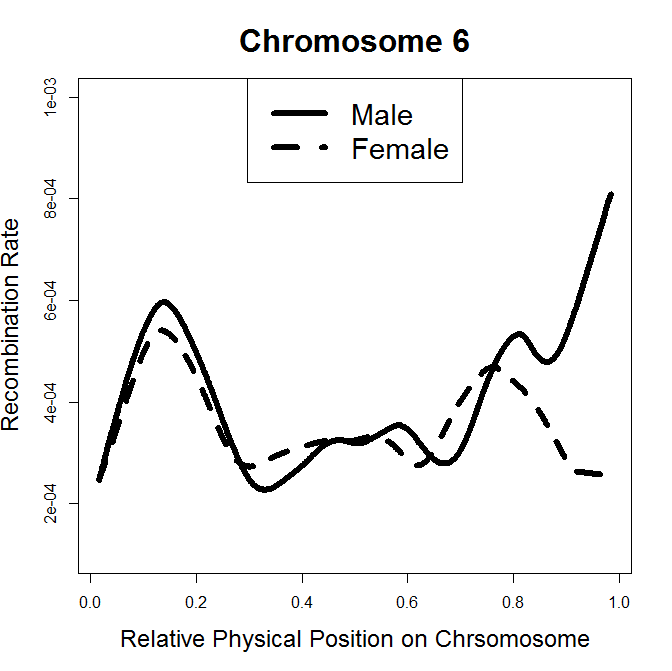

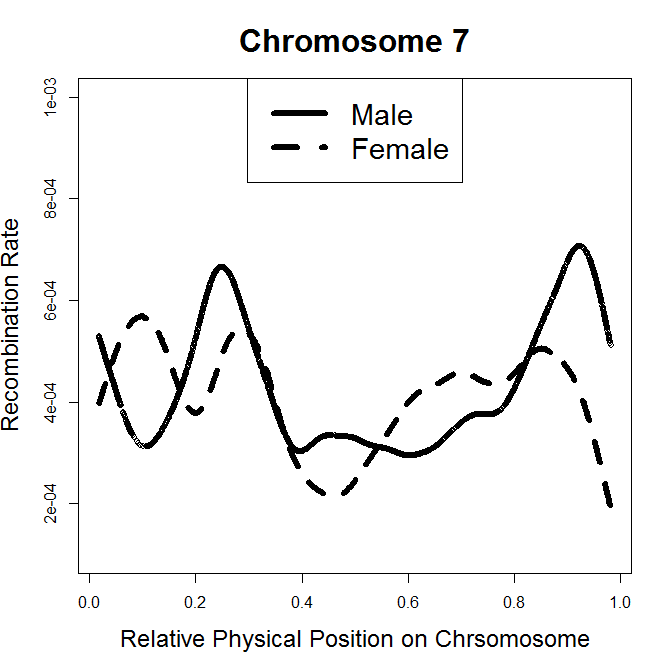

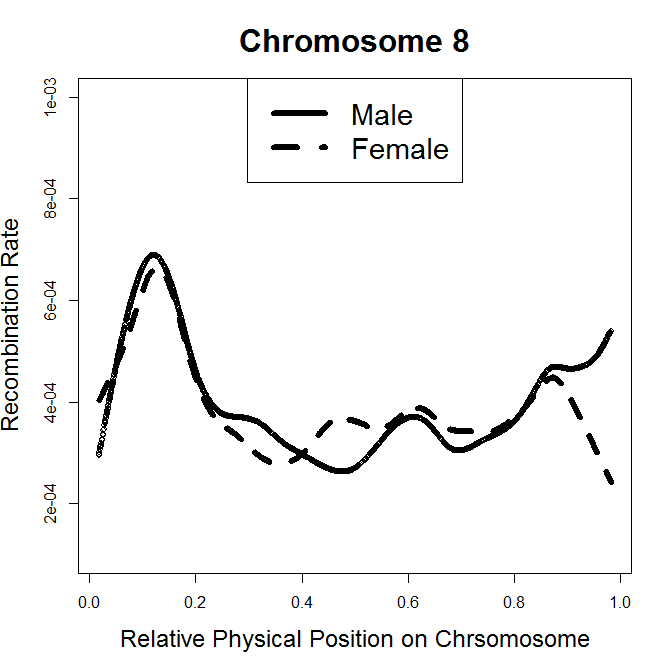

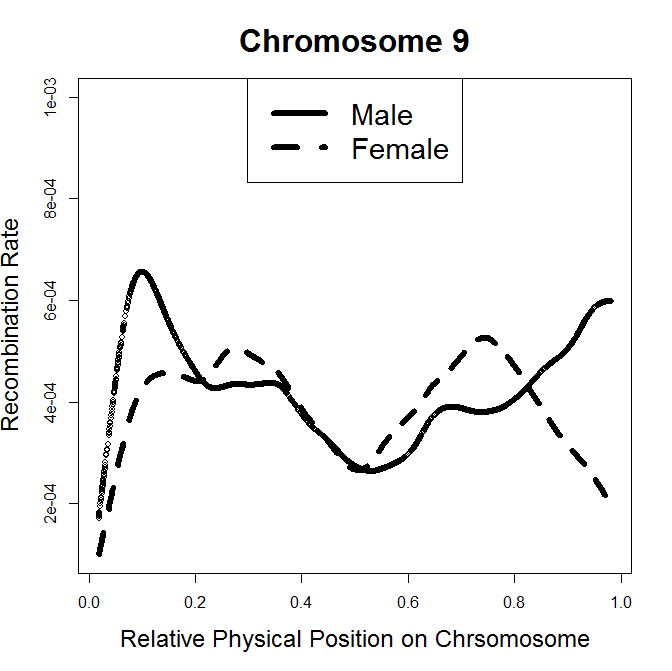

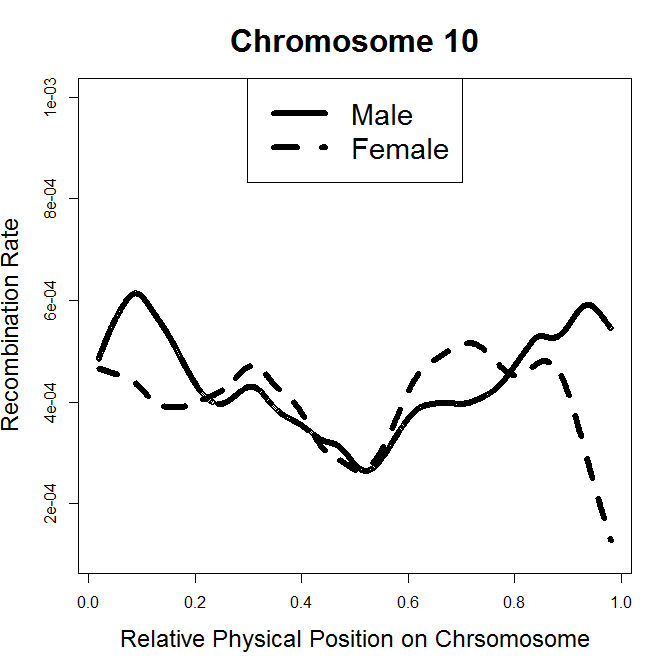

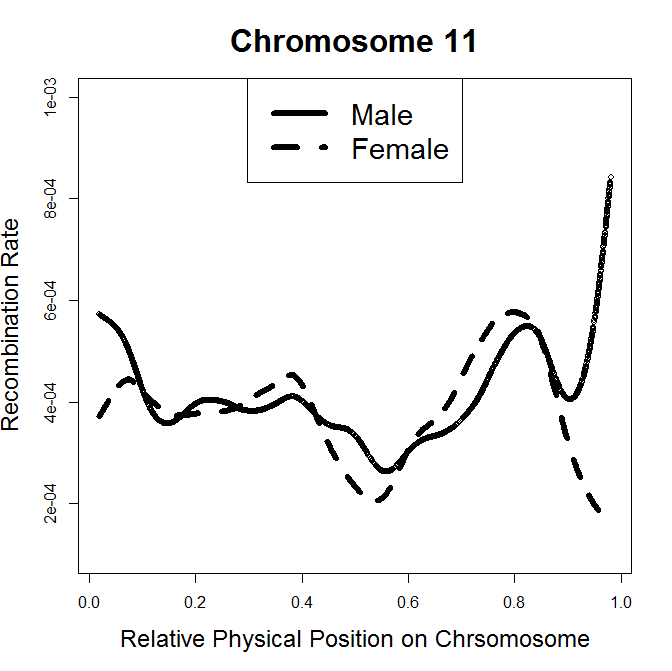

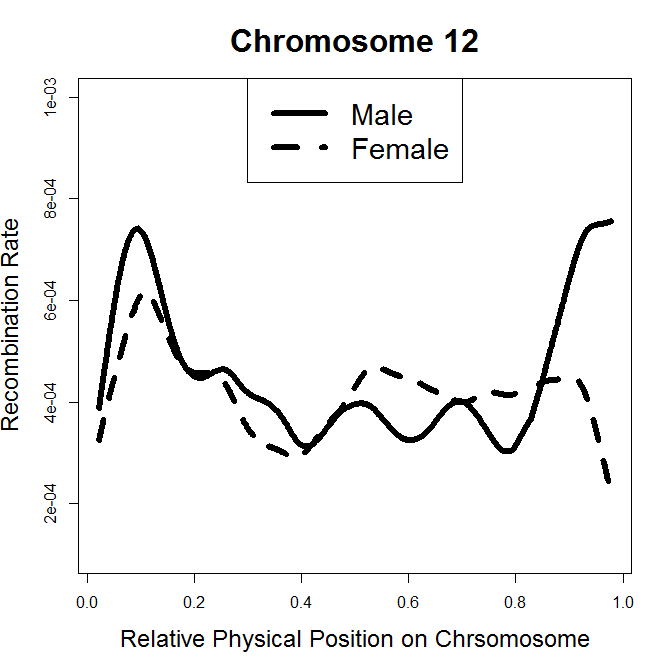

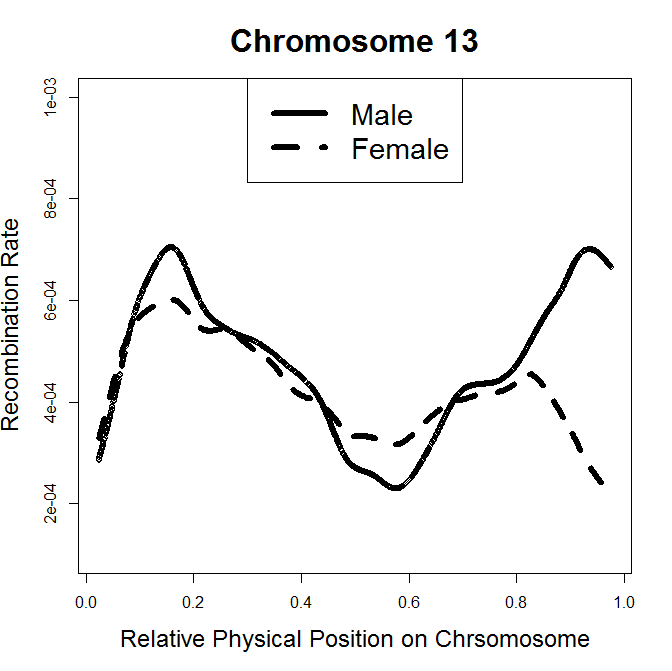

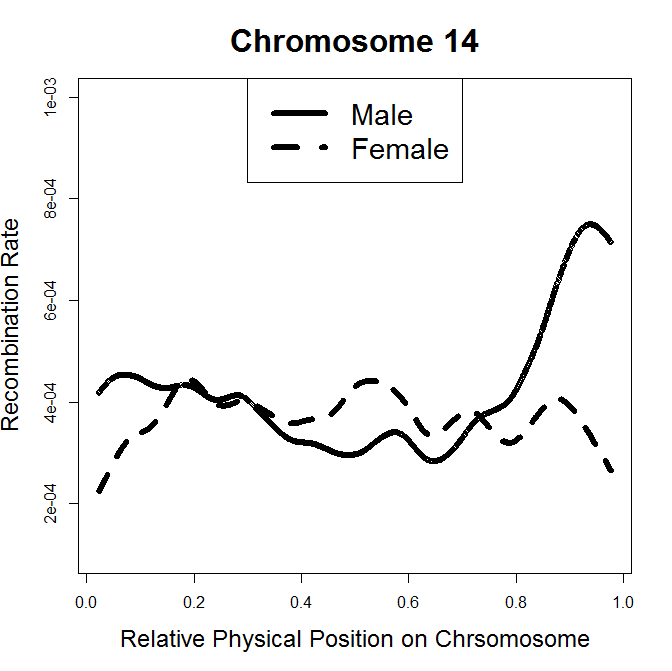

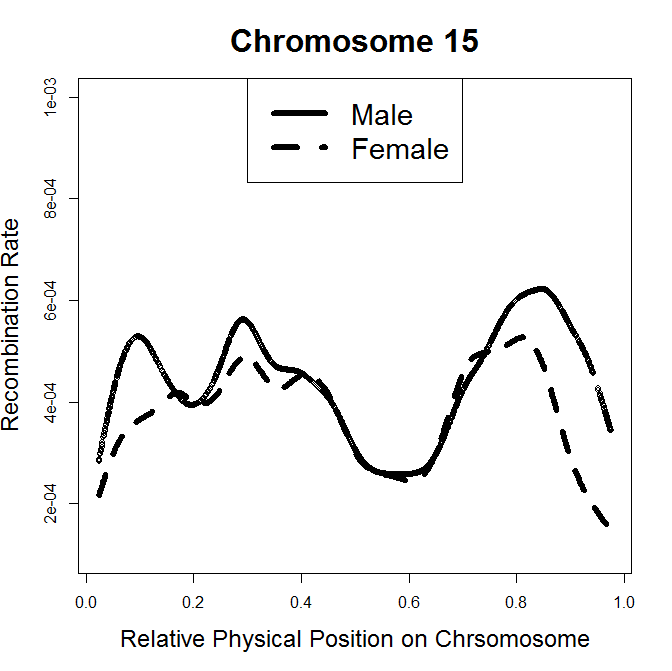

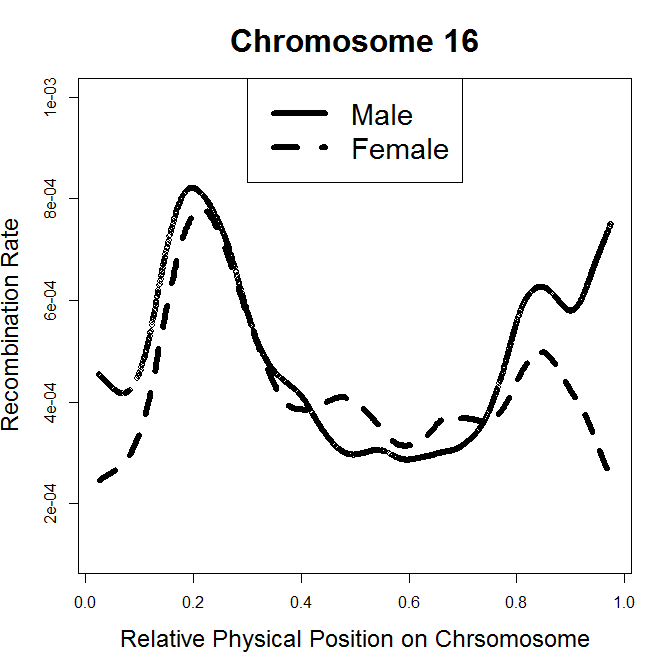

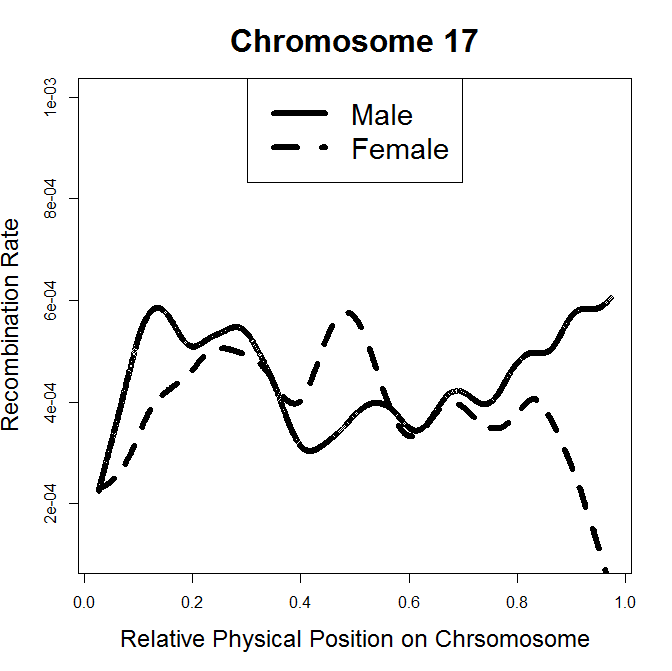

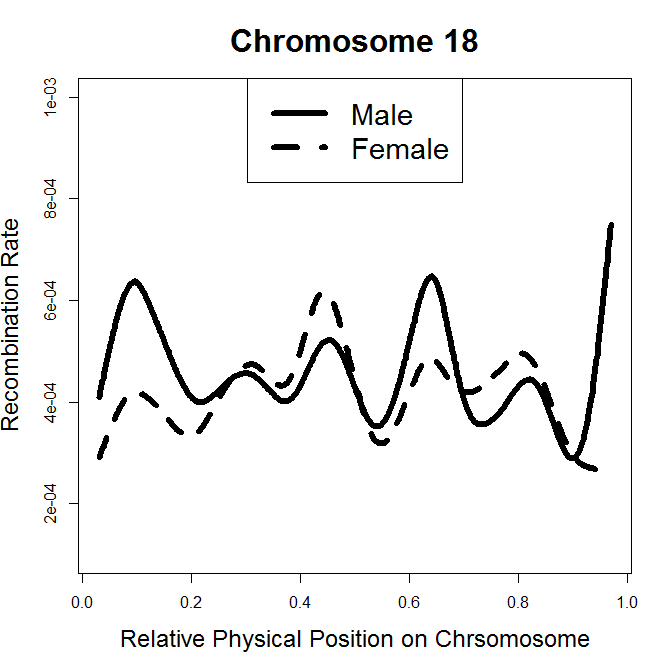

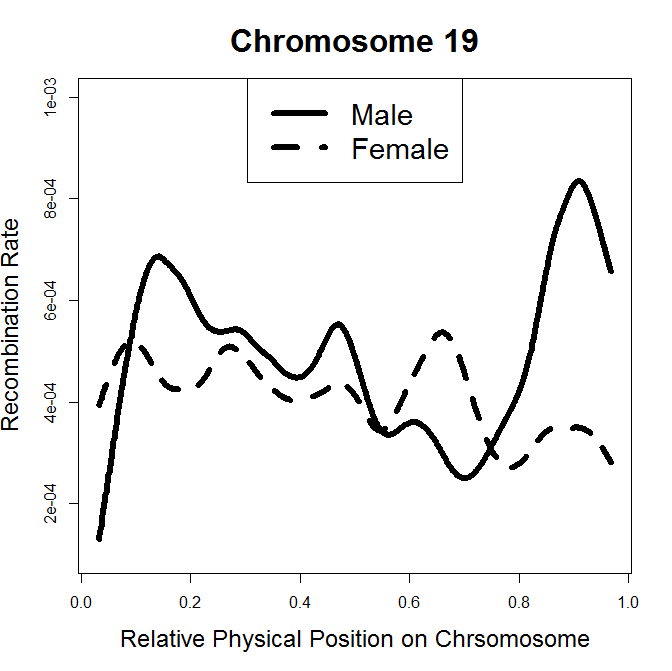

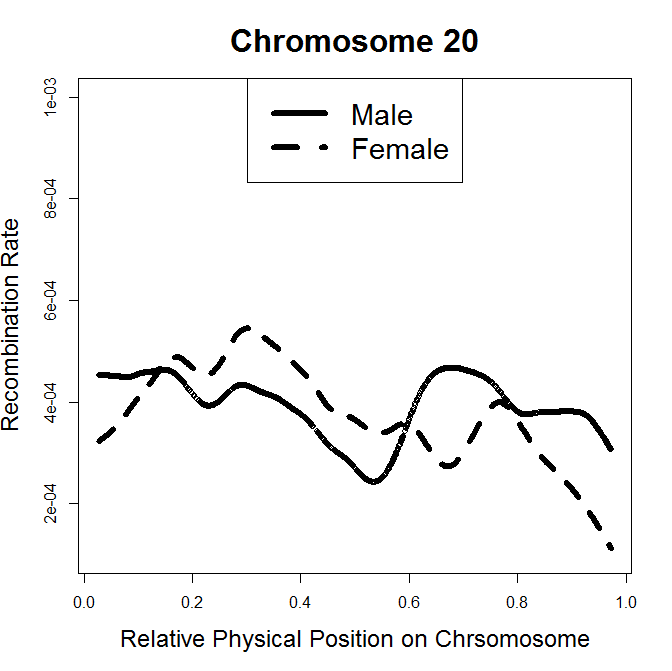

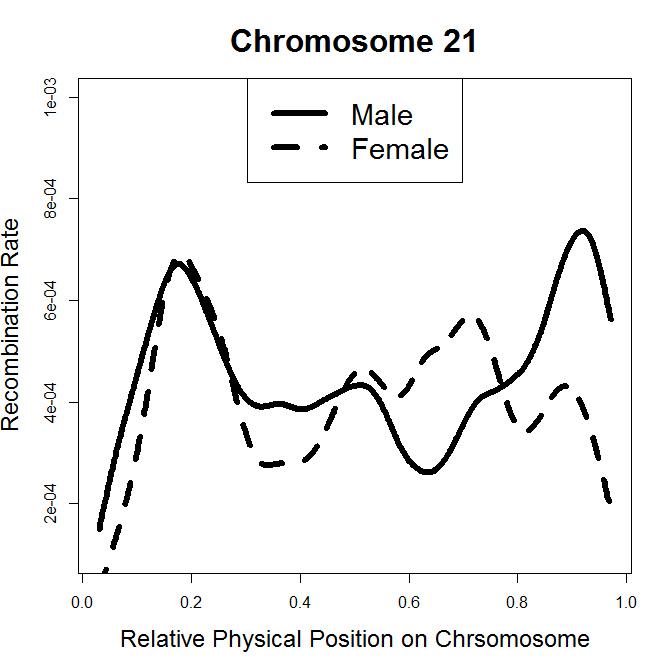

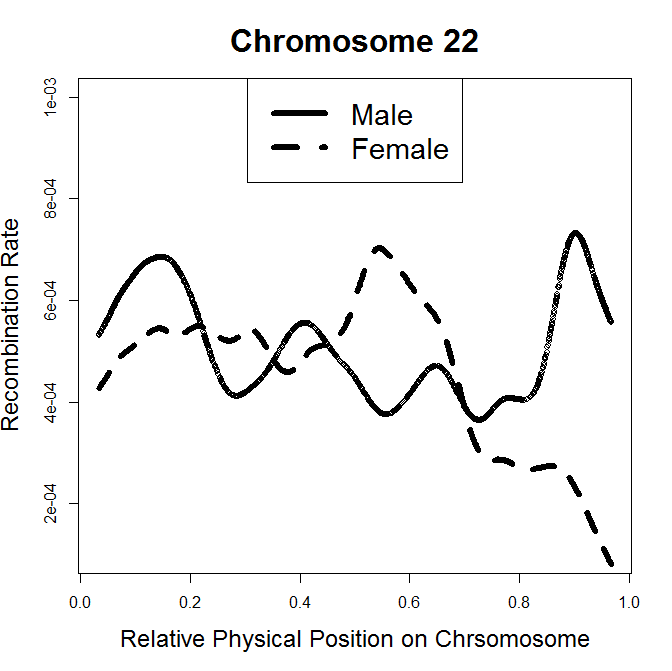

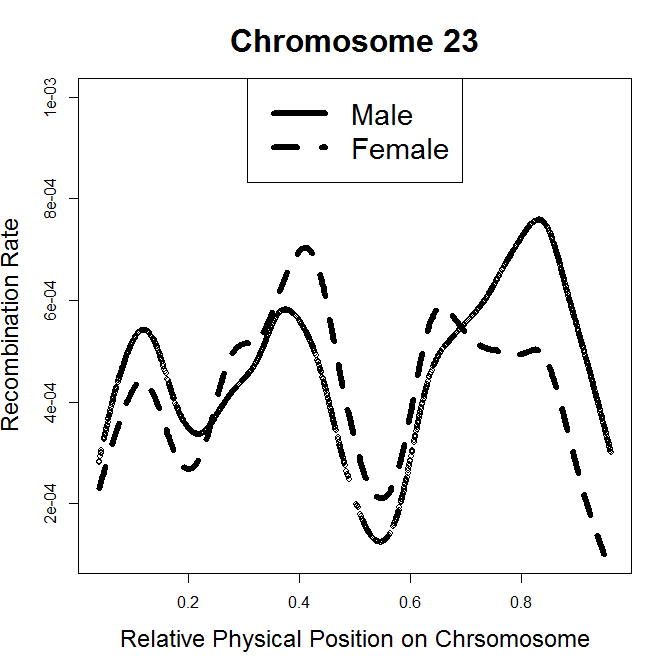

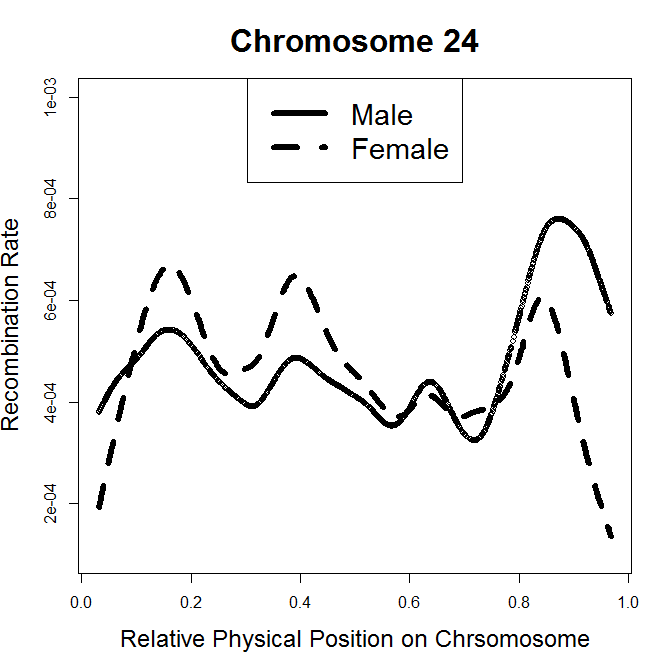


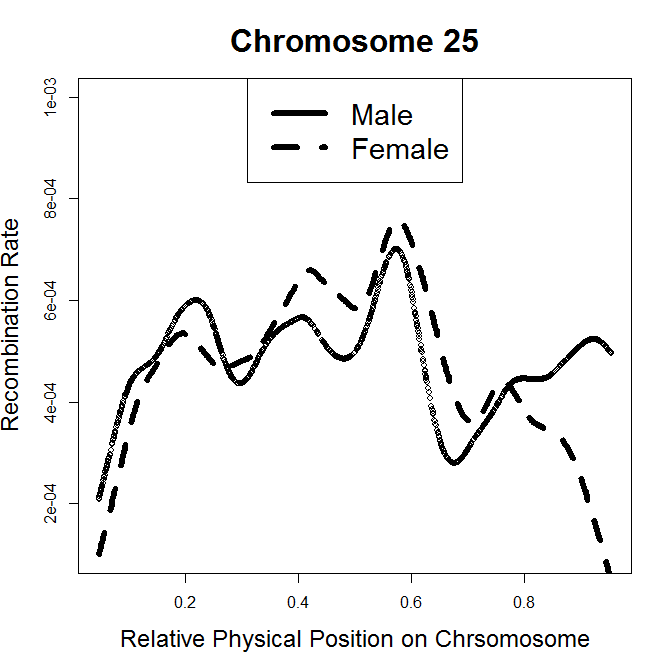

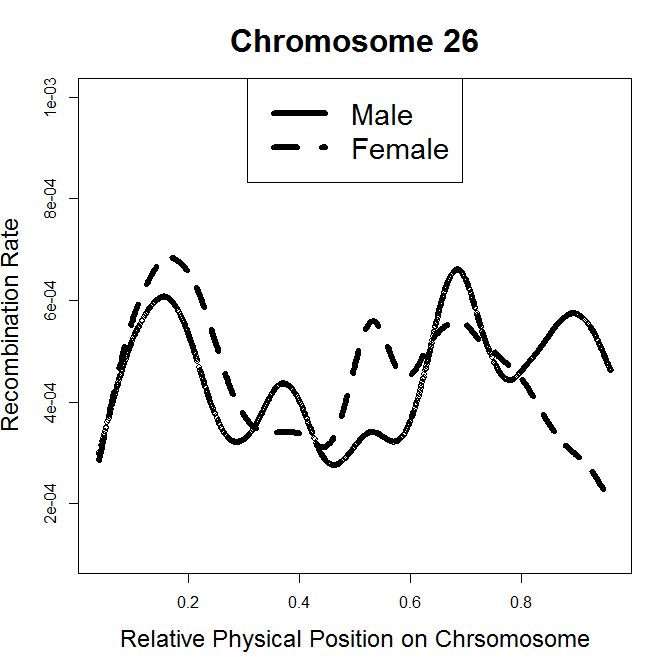

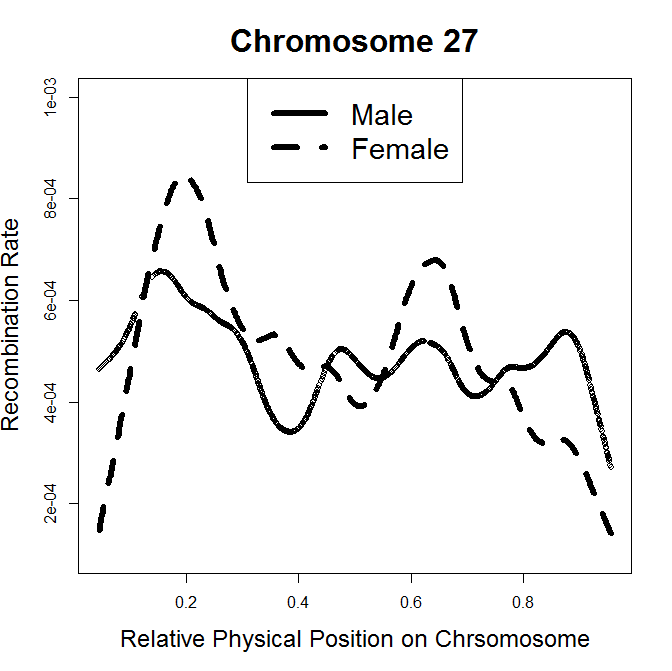


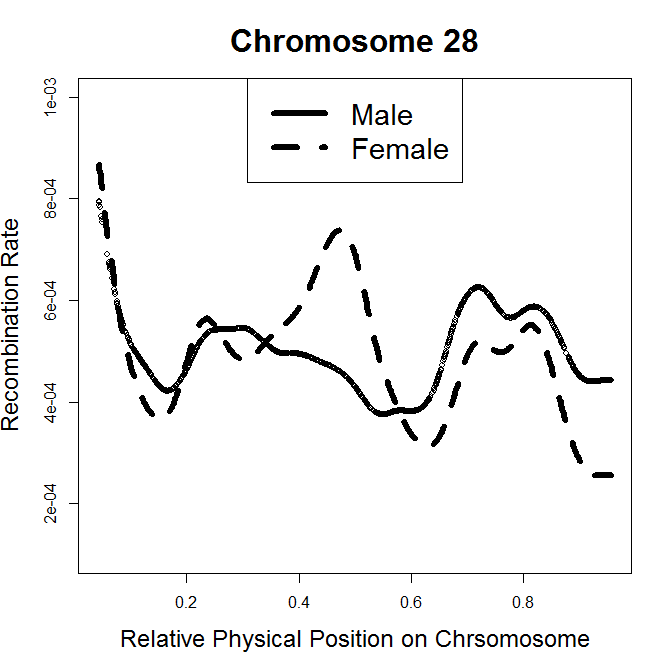

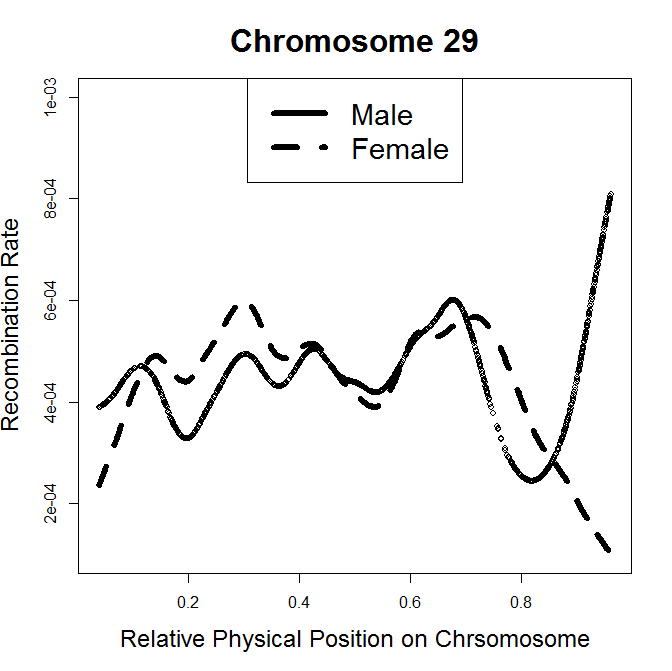

Supplement: S3 Fig — (DOCX) [file pgen.1005387.s003.docx]
