# Supplementary material for: Cattle Sex-Specific Recombination and Genetic Control from a Large Pedigree Analysis
Source: PLoS Genet. 2015 Nov 5;11(11):e1005387. doi: 10.1371/journal.pgen.1005387 (PMC4634960; doi:10.1371/journal.pgen.1005387)
Supplement: S4 Fig — (DOCX) [file pgen.1005387.s004.docx]

**Figure S4. Smooth spline plotting of recombination rate versus relative physical locations for single-crossover and double crossover meioses in the two sexes.**

**
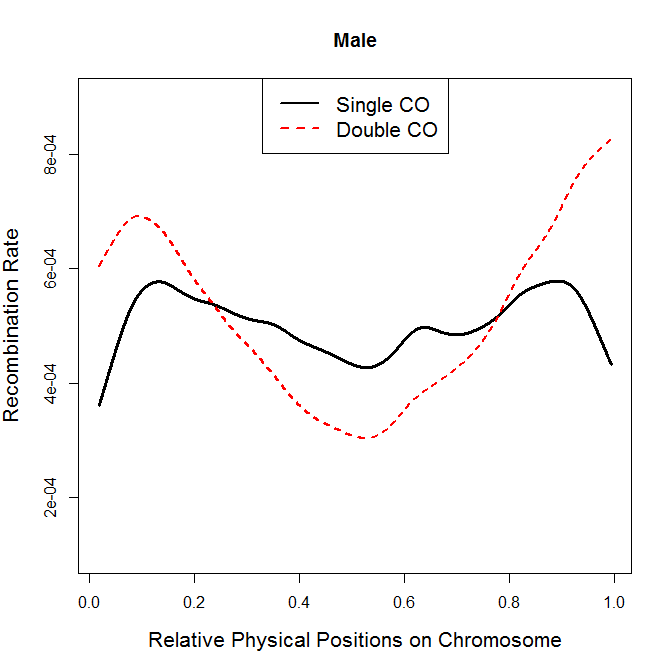

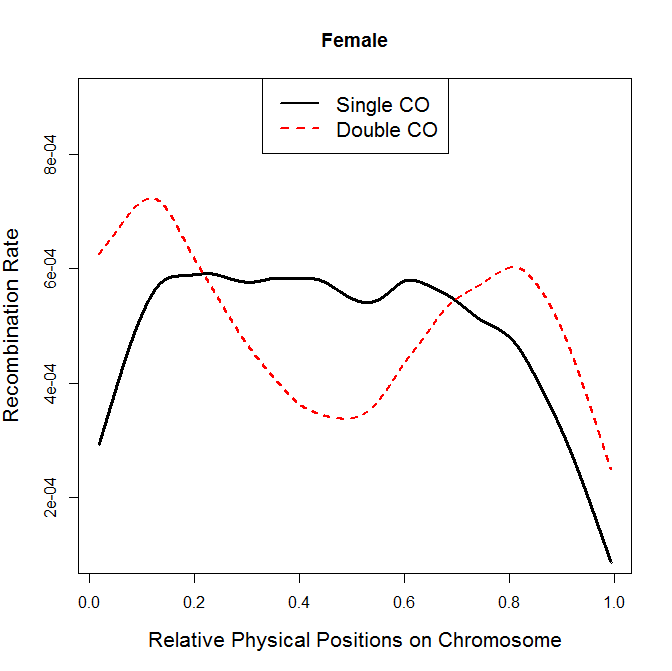
**
