# Supplementary figures and images for: Cattle Sex-Specific Recombination and Genetic Control from a Large Pedigree Analysis
Source: PLoS Genet. 2015 Nov 5;11(11):e1005387. doi: 10.1371/journal.pgen.1005387 (PMC4634960; doi:10.1371/journal.pgen.1005387)

**Figure S5. QQ-plot for the GWAS of recombination rate in males (A) and females (B).**


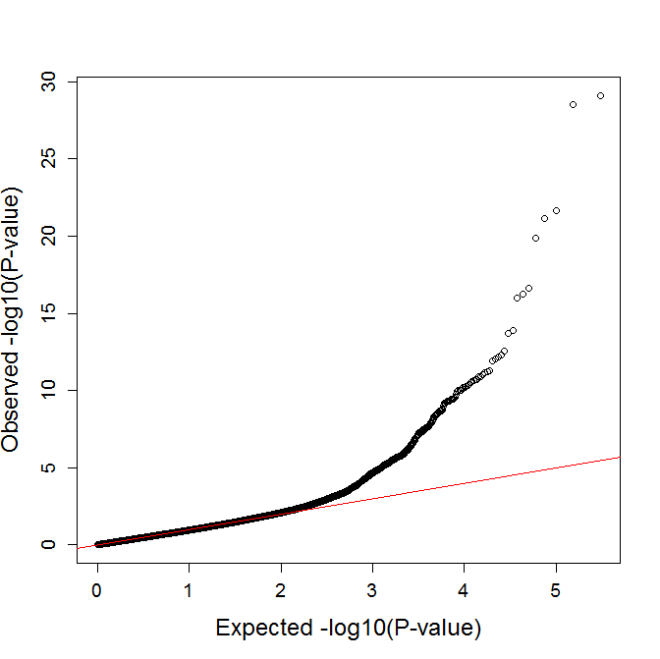

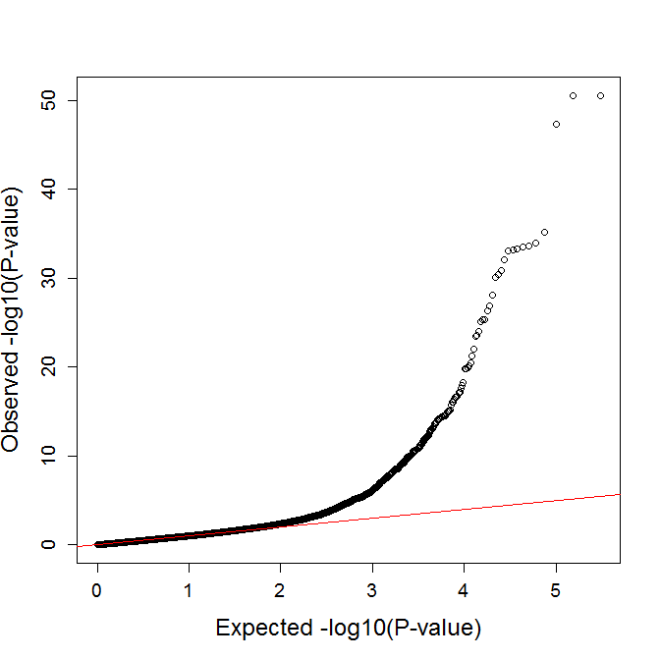


**A**

**B**

Supplement: S5 Fig — (DOCX) [file pgen.1005387.s005.docx]

**Figure S6. QQ-plot for the GWAS of hotspot usage in males (A) and females (B).**


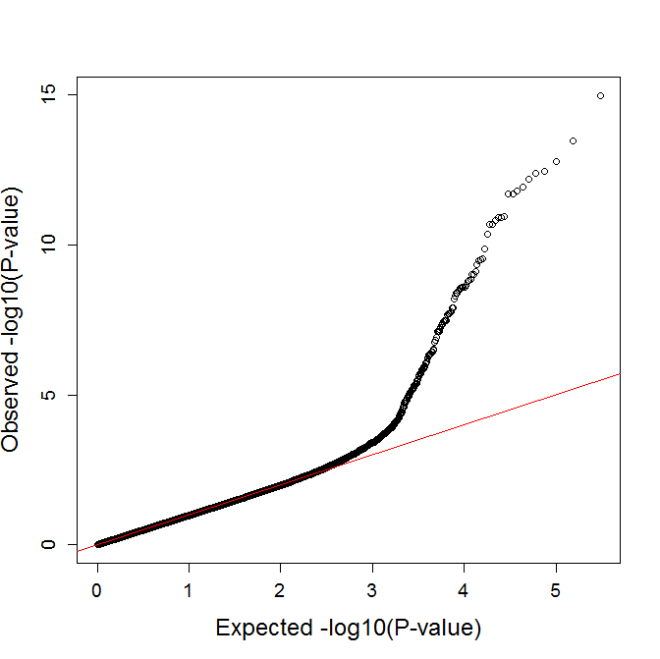

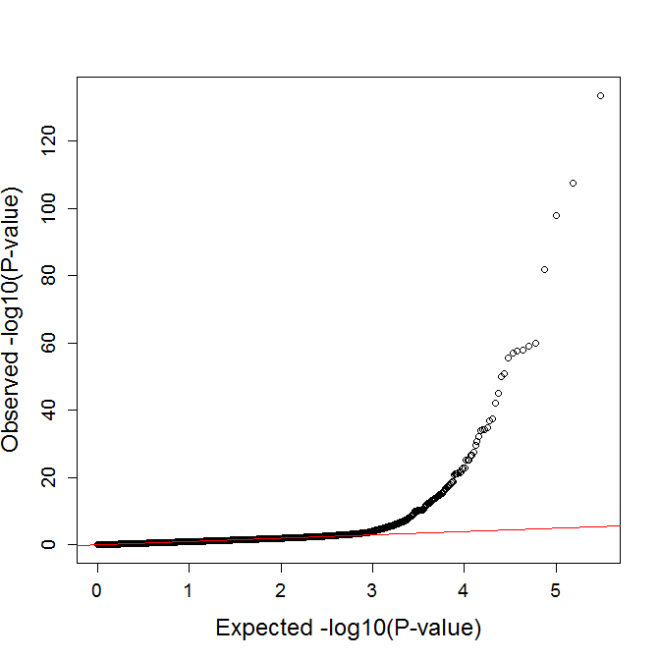


**B**

**A**

Supplement: S6 Fig — (DOCX) [file pgen.1005387.s006.docx]
