# Supplementary material for: Cattle Sex-Specific Recombination and Genetic Control from a Large Pedigree Analysis
Source: PLoS Genet. 2015 Nov 5;11(11):e1005387. doi: 10.1371/journal.pgen.1005387 (PMC4634960; doi:10.1371/journal.pgen.1005387)
Supplement: S7 Fig — (DOCX) [file pgen.1005387.s007.docx]

**Figure S7. Pairwise linkage disequilibrium patterns for LOD score (A) and recombination rate (B) between the top associated SNP, rs110661033 or ARS-BFGL-NGS-83544**, **near *PRDM9* and all other SNPs on the same chromosome.** Vertical strait line denotes the location of rs110661033.


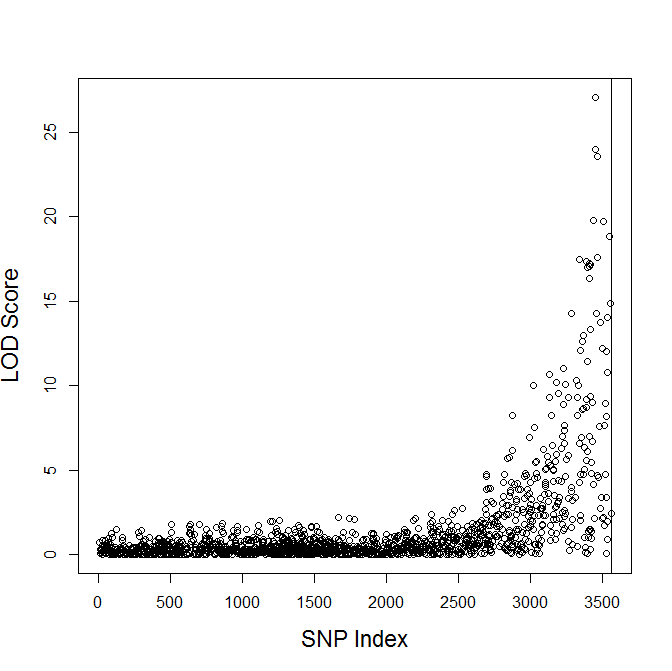

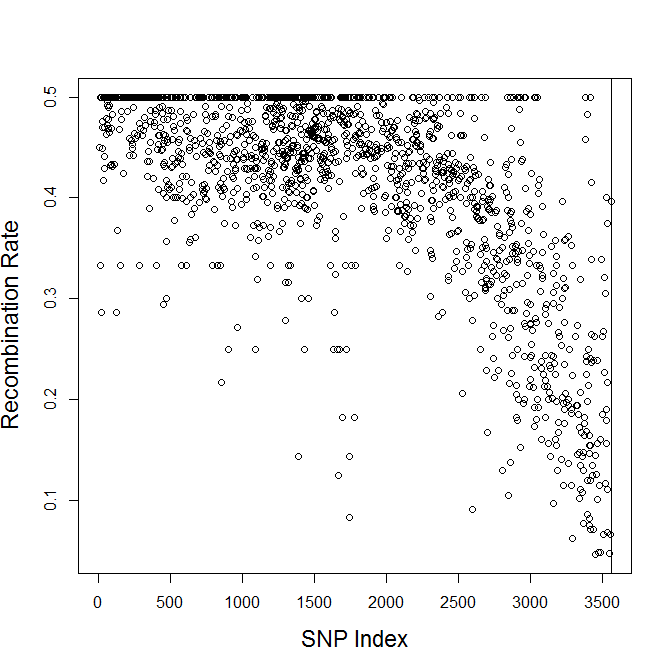


**A**

**B**
