# Supplementary material for: Cattle Sex-Specific Recombination and Genetic Control from a Large Pedigree Analysis
Source: PLoS Genet. 2015 Nov 5;11(11):e1005387. doi: 10.1371/journal.pgen.1005387 (PMC4634960; doi:10.1371/journal.pgen.1005387)
Supplement: S9 Fig — (DOCX) [file pgen.1005387.s009.docx]

**Figure S9. Time trend of hotspot usage using a scatter plot and a smooth spline in males (A) and females (B), and a zoomed-in plot after 1990 in both sexes (C).**


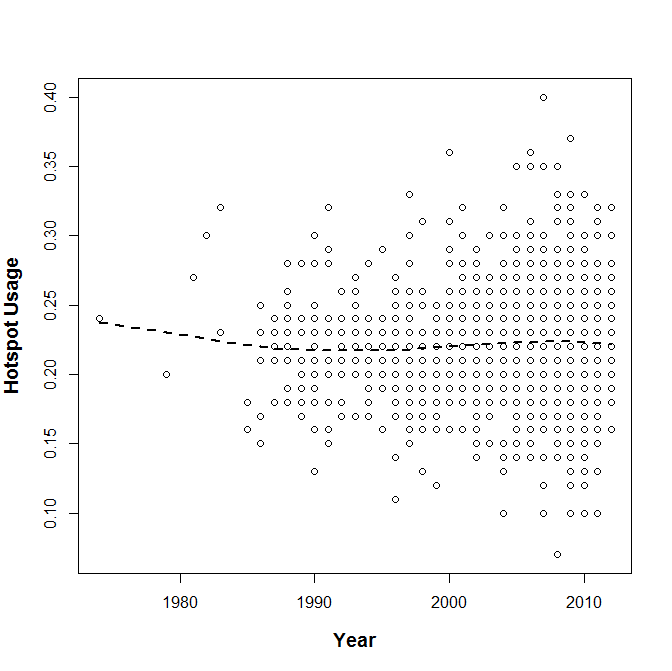

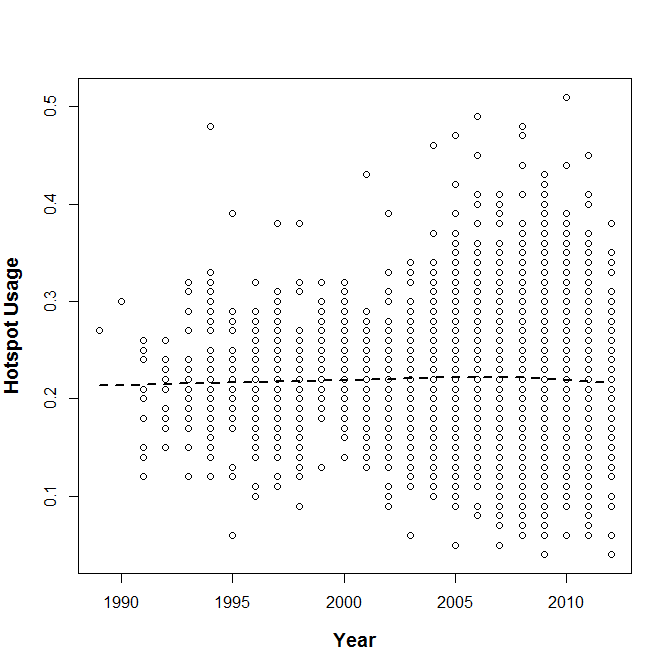


**B**

**A**


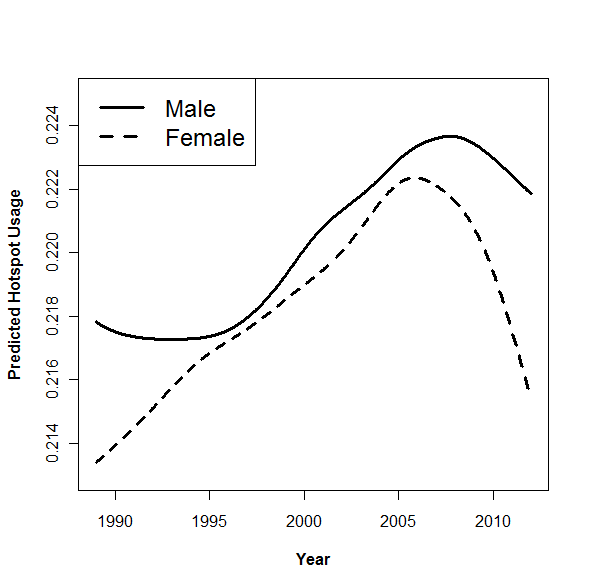


**C**
