# Supplementary material for: Cattle Sex-Specific Recombination and Genetic Control from a Large Pedigree Analysis
Source: PLoS Genet. 2015 Nov 5;11(11):e1005387. doi: 10.1371/journal.pgen.1005387 (PMC4634960; doi:10.1371/journal.pgen.1005387)
Supplement: S10 Fig — (DOCX) [file pgen.1005387.s010.docx]

**Figure S10. Smooth spline plotting of recombination rate versus relative physical locations in humans for all autosomes (A) and for acrocentric chromosomes (B), including chr13, chr14, chr15, chr21 and chr22.** The deCODE human recombination maps were used to generate the plots (<http://www.decode.com/addendum/>).

**
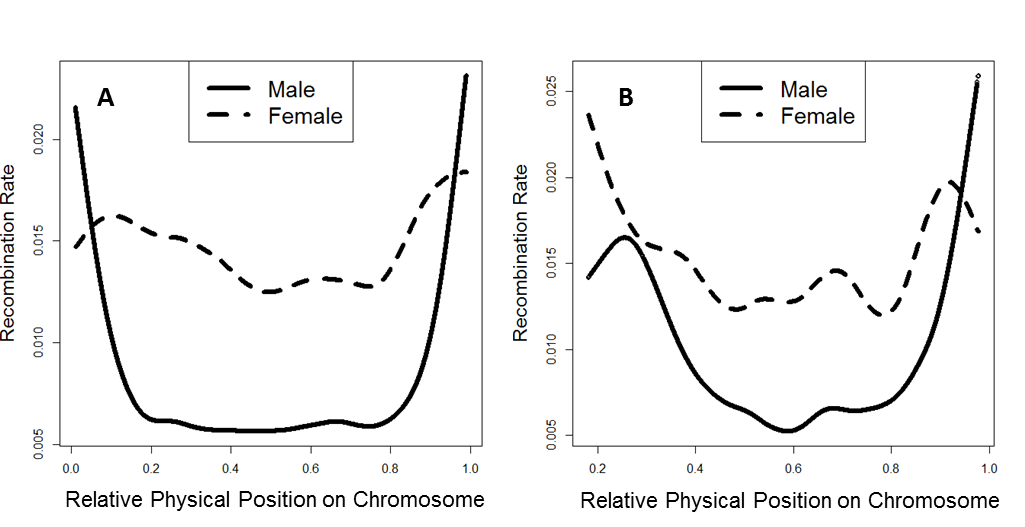
**
