# Supplementary material for: Cattle Sex-Specific Recombination and Genetic Control from a Large Pedigree Analysis
Source: PLoS Genet. 2015 Nov 5;11(11):e1005387. doi: 10.1371/journal.pgen.1005387 (PMC4634960; doi:10.1371/journal.pgen.1005387)
Supplement: S11 Fig — (DOCX) [file pgen.1005387.s011.docx]

**Figure S11. Frequency change over years for alleles with positive effects on recombination rate for the 5 SNPs in Table 1.**


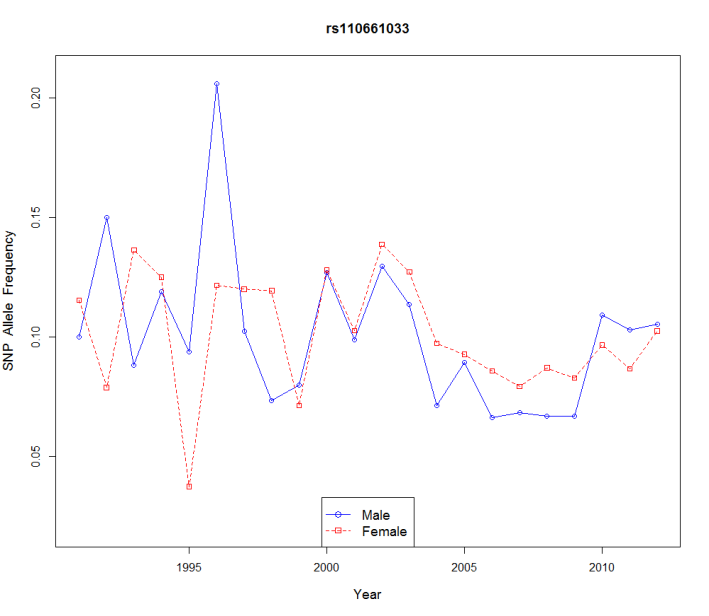

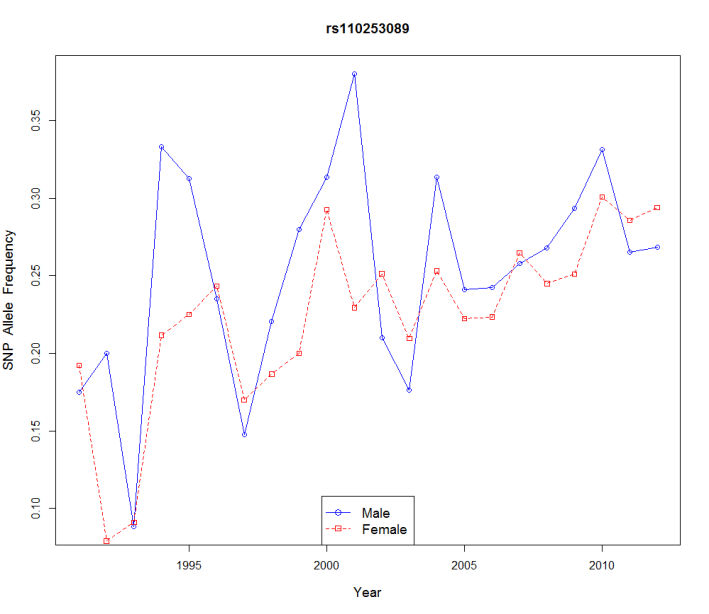

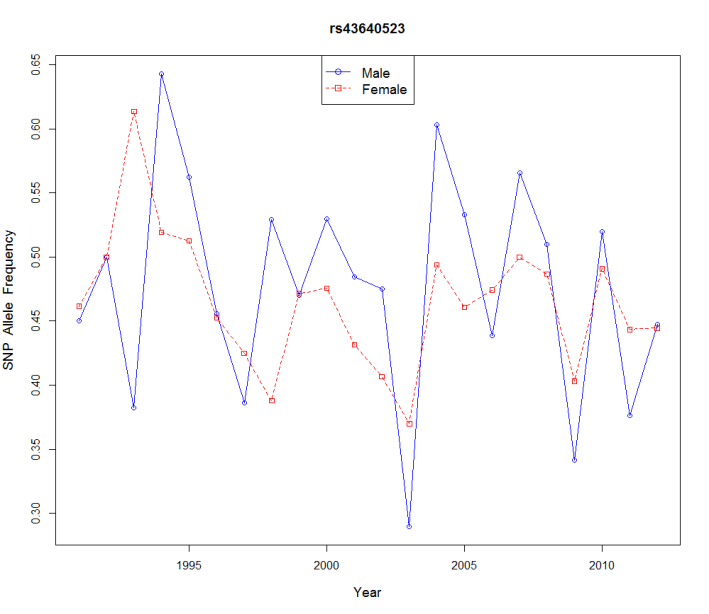

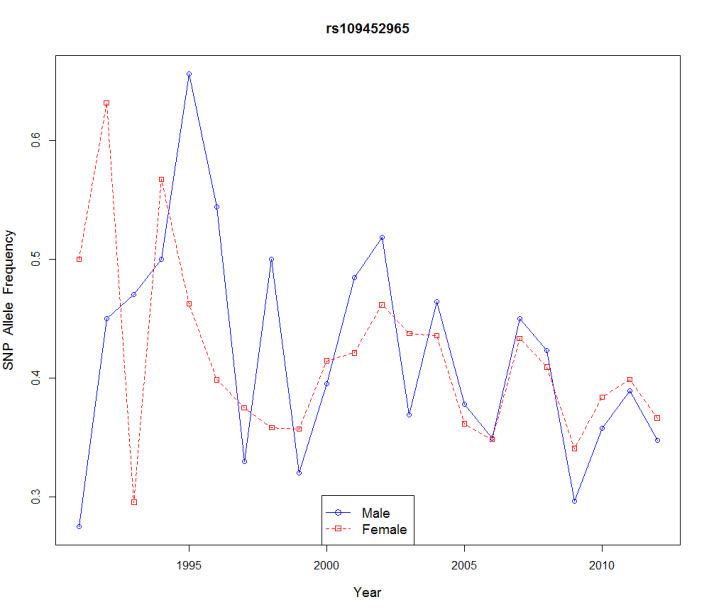
**
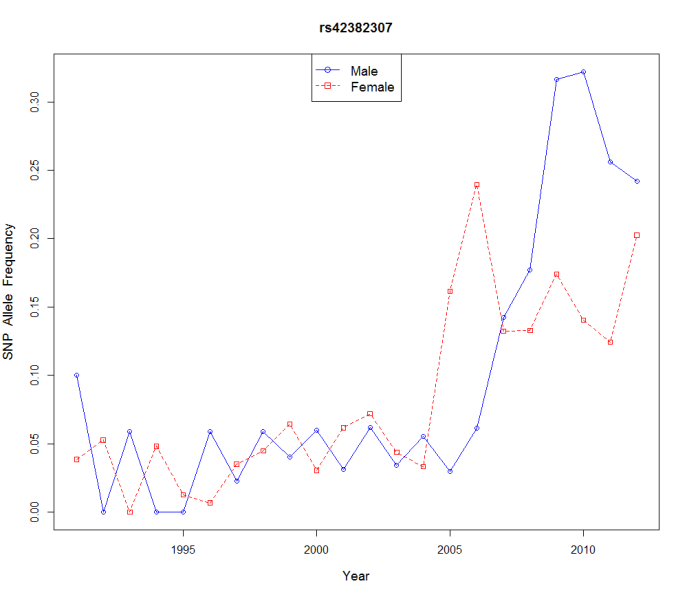
**
