# Supplementary material for: Cattle Sex-Specific Recombination and Genetic Control from a Large Pedigree Analysis
Source: PLoS Genet. 2015 Nov 5;11(11):e1005387. doi: 10.1371/journal.pgen.1005387 (PMC4634960; doi:10.1371/journal.pgen.1005387)
Supplement: S12 Fig — (DOCX) [file pgen.1005387.s012.docx]

**Figure S12. Smooth spline plotting of recombination rate per 100 kb distance versus relative physical locations with correction for physical distance of each SNP interval.**

**
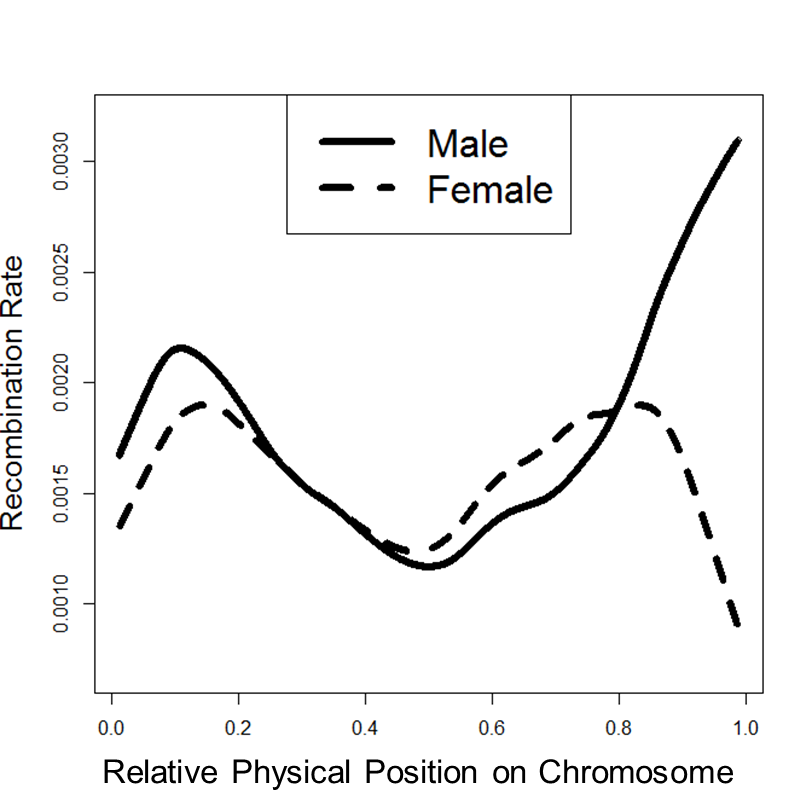
**
