# Supplementary material for: Cattle Sex-Specific Recombination and Genetic Control from a Large Pedigree Analysis
Source: PLoS Genet. 2015 Nov 5;11(11):e1005387. doi: 10.1371/journal.pgen.1005387 (PMC4634960; doi:10.1371/journal.pgen.1005387)

**Figure S13. Manhattan plots for the GWAS of hotspot usage with correction for physical distance of each SNP interval in males and females.** Different colors were used to distinguish the 29 chromosomes. The genome-wide significance level of 1.6×10^-7^ was shown by the horizontal dotted line. We selected 2,875 male and 3,005 female hotspots for which the standardized recombination rate is 0.6 standard deviations higher than the mean. This cutoff of 0.6 standard deviations identified hotspots that accounted for ~25% of all recombination events.


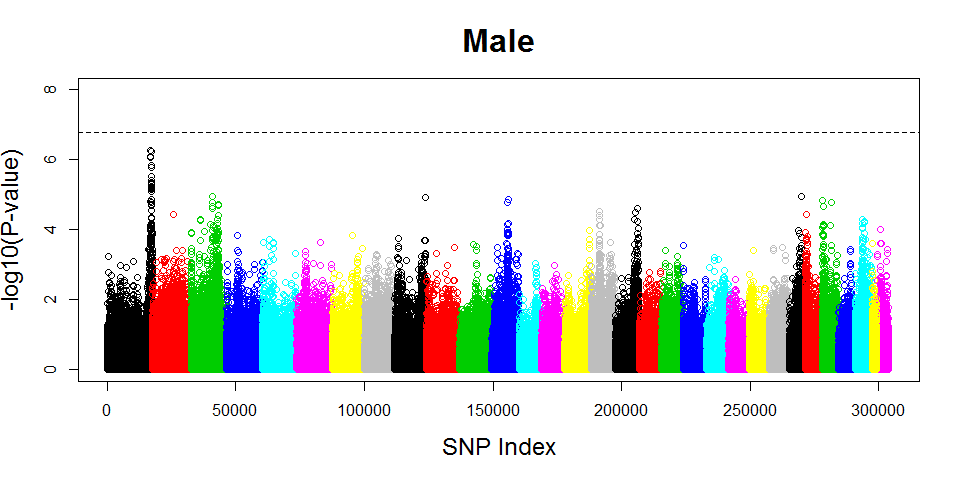

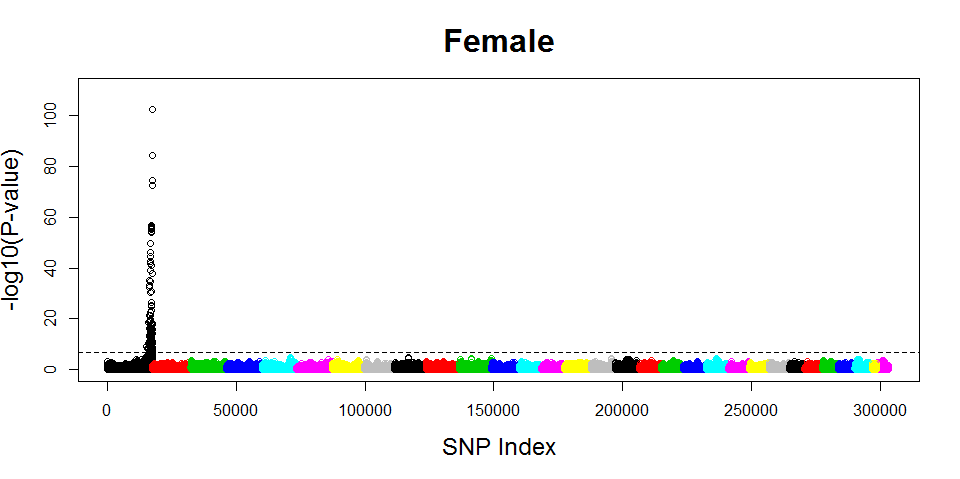

Supplement: S13 Fig — (DOCX) [file pgen.1005387.s013.docx]
