# Supplementary material for: Cattle Sex-Specific Recombination and Genetic Control from a Large Pedigree Analysis
Source: PLoS Genet. 2015 Nov 5;11(11):e1005387. doi: 10.1371/journal.pgen.1005387 (PMC4634960; doi:10.1371/journal.pgen.1005387)
Supplement: S1 Table — (DOCX) [file pgen.1005387.s014.docx]

**Table S1. SNP chips, the number of SNPs and the number of genotyped animals used in this study.** The animals were genotyped by various chips, including the Illumina Bovine High-Density (HD) chip, Geneseek HD chip, BovineSNP50 v1 and v2 chips, the Zoetis BovineLD chip, the GeneSeek Genomic Profiler chips, the Illumina BovineLD BeadChip, the Illumina Bovine3K BeadChip. The SNP chips with more than 50K SNPs were combined into one category and referred to as the 50K chip.

| **SNP Chip** | **Number of SNPs** | **Referred Name** | **Genotyped Bulls** | **Genotyped Cows** |
| --- | --- | --- | --- | --- |
| Bovine High-Density chip | 777,962 | 50K | 92 | 261 |
| GeneSeek HD chip | 77,068 | 50K | 7489 | 8262 |
| BovineSNP50 v1 | 56,947 | 50K | 11973 | 14299 |
| BovineSNP50 v2 | 54,609 | 50K | 22242 | 19315 |
| Zoetis BovineLD chip | 10,555 | 10K | 189 | 9676 |
| GeneSeek Genomic Profiler 2 | 8,415 | 8K | 17149 | 26176 |
| GeneSeek Genomic Profiler | 8,042 | 8K | 8620 | 19870 |
| Illumina BovineLD BeadChip | 6,785 | 7K | 2135 | 32678 |
| Illumina Bovine3K BeadChip | 2,708 | 3K | 1467 | 21471 |
| **Total** |  |  | **71,356** | **152,008** |
