# Supplementary material for: Cattle Sex-Specific Recombination and Genetic Control from a Large Pedigree Analysis
Source: PLoS Genet. 2015 Nov 5;11(11):e1005387. doi: 10.1371/journal.pgen.1005387 (PMC4634960; doi:10.1371/journal.pgen.1005387)
Supplement: S2 Table — (DOCX) [file pgen.1005387.s015.docx]

**Table S2. The number of recombination events identified and SNP chips used for paternal meioses.** The total number recombination events identified depends on the number of SNPs genotyped for the offspring, the parent, and the grandparents in a three-generation family. Categories with a sample size less than 10 were not shown.

| **Chip_Offspring** | **Chip_Sire** | **Chip_Grandsire** | **Chip_Granddam** | **#Crossover** | **#Meioses** |
| --- | --- | --- | --- | --- | --- |
| 50K | 50K | 50K | 50K | 25.5 | 38031 |
| 50K | 50K | 50K | 7K | 24.4 | 220 |
| 50K | 50K | 50K | 3K | 25.9 | 760 |
| 50K | 50K | 50K | 0 | 25.1 | 31704 |
| 50K | 7K | 50K | 50K | 24.9 | 12 |
| 10K | 50K | 50K | 50K | 24.8 | 6265 |
| 10K | 50K | 50K | 3K | 24.7 | 99 |
| 10K | 50K | 50K | 0 | 24.4 | 3018 |
| 8K | 50K | 50K | 50K | 24.6 | 35415 |
| 8K | 50K | 50K | 8K | 24.9 | 18 |
| 8K | 50K | 50K | 7K | 25.1 | 51 |
| 8K | 50K | 50K | 3K | 25.1 | 1669 |
| 8K | 50K | 50K | 0 | 23.3 | 5380 |
| 8K | 7K | 50K | 50K | 25.3 | 15 |
| 8K | 50K | 50K | 50K | 24.3 | 18411 |
| 8K | 50K | 50K | 7K | 23.5 | 37 |
| 8K | 50K | 50K | 3K | 23.8 | 172 |
| 8K | 50K | 50K | 0 | 23.1 | 7334 |
| 8K | 50K | 50K | 50K | 24.8 | 74 |
| 8K | 50K | 50K | 0 | 22.9 | 20 |
| 7K | 50K | 50K | 50K | 24.3 | 13832 |
| 7K | 50K | 50K | 7K | 23.7 | 48 |
| 7K | 50K | 50K | 3K | 24.5 | 256 |
| 7K | 50K | 50K | 0 | 23.1 | 9110 |
| 7K | 8K | 50K | 0 | 21.5 | 14 |
| 7K | 7K | 50K | 50K | 23.4 | 13 |
| 3K | 50K | 50K | 50K | 20.5 | 5132 |
| 3K | 50K | 50K | 7K | 19.0 | 106 |
| 3K | 50K | 50K | 0 | 17.7 | 8575 |
