# Supplementary material for: Cattle Sex-Specific Recombination and Genetic Control from a Large Pedigree Analysis
Source: PLoS Genet. 2015 Nov 5;11(11):e1005387. doi: 10.1371/journal.pgen.1005387 (PMC4634960; doi:10.1371/journal.pgen.1005387)
Supplement: S3 Table — (DOCX) [file pgen.1005387.s016.docx]

**Table S3. The number of recombination events identified and SNP chips used for maternal meioses.** The total number recombination events identified depends on the number of SNPs genotyped for the offspring, the parent, and the grandparents in a three-generation family. Categories with a sample size less than 10 were not shown.

| **Chip_Offspring** | **Chip_Dam** | **Chip_Grandsire** | **Chip_Granddam** | **#Crossover** | **#Meioses** |
| --- | --- | --- | --- | --- | --- |
| 50K | 50K | 50K | 50K | 23.2 | 30046 |
| 50K | 50K | 50K | 8K | 23.2 | 102 |
| 50K | 50K | 50K | 7K | 23.9 | 72 |
| 50K | 50K | 50K | 3K | 23.2 | 803 |
| 50K | 50K | 50K | 0 | 22.6 | 30593 |
| 50K | 8K | 50K | 50K | 21.3 | 64 |
| 50K | 8K | 50K | 0 | 22.3 | 127 |
| 50K | 8K | 50K | 50K | 22.6 | 365 |
| 50K | 8K | 50K | 8K | 21.6 | 42 |
| 50K | 8K | 50K | 3K | 22.4 | 69 |
| 50K | 8K | 50K | 0 | 21.7 | 477 |
| 50K | 7K | 50K | 50K | 22.1 | 406 |
| 50K | 7K | 50K | 7K | 23.8 | 59 |
| 50K | 7K | 50K | 3K | 21.4 | 83 |
| 50K | 7K | 50K | 0 | 21.5 | 618 |
| 50K | 3K | 50K | 50K | 20.0 | 2711 |
| 50K | 3K | 50K | 8K | 20.1 | 31 |
| 50K | 3K | 50K | 7K | 18.2 | 30 |
| 50K | 3K | 50K | 3K | 20.0 | 521 |
| 50K | 3K | 50K | 0 | 17.4 | 3492 |
| 10K | 50K | 50K | 50K | 22.8 | 436 |
| 10K | 50K | 50K | 0 | 22.3 | 256 |
| 10K | 10K | 50K | 0 | 22.2 | 328 |
| 10K | 8K | 50K | 50K | 22.1 | 87 |
| 10K | 8K | 50K | 3K | 22.4 | 24 |
| 10K | 8K | 50K | 0 | 21.9 | 499 |
| 10K | 7K | 50K | 50K | 21.9 | 197 |
| 10K | 7K | 50K | 7K | 22.1 | 115 |
| 10K | 7K | 50K | 3K | 22.6 | 150 |
| 10K | 7K | 50K | 0 | 21.7 | 6214 |
| 10K | 3K | 50K | 50K | 19.5 | 168 |
| 10K | 3K | 50K | 3K | 19.1 | 48 |
| 10K | 3K | 50K | 0 | 17.5 | 762 |
| 8K | 50K | 50K | 50K | 22.6 | 24702 |
| 8K | 50K | 50K | 8K | 22.3 | 92 |
| 8K | 50K | 50K | 7K | 22.0 | 46 |
| 8K | 50K | 50K | 3K | 22.3 | 1206 |
| 8K | 50K | 50K | 0 | 21.5 | 13386 |
| 8K | 8K | 50K | 50K | 22.7 | 2789 |
| 8K | 8K | 50K | 8K | 23.0 | 201 |
| 8K | 8K | 50K | 7K | 22.7 | 51 |
| 8K | 8K | 50K | 3K | 22.3 | 708 |
| 8K | 8K | 50K | 0 | 21.6 | 3904 |
| 8K | 7K | 50K | 50K | 22.3 | 2066 |
| 8K | 7K | 50K | 8K | 22.2 | 66 |
| 8K | 7K | 50K | 7K | 22.1 | 150 |
| 8K | 7K | 50K | 3K | 22.0 | 754 |
| 8K | 7K | 50K | 0 | 21.6 | 2528 |
| 8K | 3K | 50K | 50K | 19.7 | 6384 |
| 8K | 3K | 50K | 8K | 20.1 | 174 |
| 8K | 3K | 50K | 7K | 20.4 | 94 |
| 8K | 3K | 50K | 3K | 19.8 | 1427 |
| 8K | 3K | 50K | 0 | 17.1 | 7685 |
| 8K | 50K | 50K | 50K | 22.7 | 26 |
| 8K | 50K | 50K | 0 | 22.5 | 58 |
| 7K | 50K | 50K | 50K | 22.4 | 5291 |
| 7K | 50K | 50K | 7K | 23.7 | 25 |
| 7K | 50K | 50K | 3K | 21.6 | 176 |
| 7K | 50K | 50K | 0 | 21.5 | 4649 |
| 7K | 10K | 50K | 0 | 22.3 | 74 |
| 7K | 8K | 50K | 50K | 22.8 | 110 |
| 7K | 8K | 50K | 3K | 20.7 | 26 |
| 7K | 8K | 50K | 0 | 21.4 | 459 |
| 7K | 7K | 50K | 50K | 22.2 | 365 |
| 7K | 7K | 50K | 7K | 21.9 | 154 |
| 7K | 7K | 50K | 3K | 21.7 | 122 |
| 7K | 7K | 50K | 0 | 21.7 | 5199 |
| 7K | 3K | 50K | 50K | 19.2 | 1608 |
| 7K | 3K | 50K | 7K | 18.3 | 26 |
| 7K | 3K | 50K | 3K | 18.9 | 471 |
| 7K | 3K | 50K | 0 | 16.0 | 4363 |
| 3K | 50K | 50K | 50K | 19.7 | 3774 |
| 3K | 50K | 50K | 3K | 19.7 | 127 |
| 3K | 50K | 50K | 0 | 17.3 | 6671 |
| 3K | 8K | 50K | 0 | 17.3 | 190 |
| 3K | 7K | 50K | 0 | 15.9 | 103 |
| 3K | 3K | 50K | 50K | 20.1 | 469 |
| 3K | 3K | 50K | 3K | 20.0 | 143 |
| 3K | 3K | 50K | 0 | 17.5 | 2237 |
