# Supplementary material for: Cattle Sex-Specific Recombination and Genetic Control from a Large Pedigree Analysis
Source: PLoS Genet. 2015 Nov 5;11(11):e1005387. doi: 10.1371/journal.pgen.1005387 (PMC4634960; doi:10.1371/journal.pgen.1005387)
Supplement: S4 Table — (DOCX) [file pgen.1005387.s017.docx]

**Table S4. SNPs associated with subtelomeric recombination rate in females and males.**

| **SNP_rs** | **SNP** | **Chr** | **Position^a^** | **Female** | | | **Male** | | | **Gene^c^** |
| --- | --- | --- | --- | --- | --- | --- | --- | --- | --- | --- |
|  |  |  |  | **Freq** | **Beta^b^** | **P-val** | **Freq** | **Beta^b^** | **P-val** |  |
| rs110661033 | ARS-BFGL-NGS-83544 | 1 | 158140250 | 0.09 | 0.30 | 4.5×10^-28^ | 0.09 | 0.31 | 4.7×10^-06^ | *PRDM9* |
| rs110253089 | ARS-BFGL-NGS-117763 | 6 | 129108015 | 0.26 | 0.15 | 2.0×10^-14^ | 0.27 | 0.27 | 2.5×10^-08^ | *CPLX1* |
|  | Hapmap47676-BTA-61231 | 10 | 21604877 | 0.14 | 0.18 | 3.5×10^-12^ | 0.13 | 0.26 | 1.5×10^-05^ | *ACIN1* |
| rs110500644 | ARS-BFGL-NGS-19822 | 10 | 22128700 | 0.41 | 0.09 | 1.9×10^-07^ | 0.42 | 0.16 | 3.7×10^-04^ | / |
| rs133086206 | BovineHD1000008512 | 10 | 26151175 | 0.44 | 0.08 | 1.6×10^-05^ | 0.42 | 0.23 | 1.8×10^-07^ | *SLC39A2* |
| rs110000217 | ARS-BFGL-NGS-118210 | 10 | 26700563 | 0.81 | -0.16 | 1.4×10^-09^ | 0.82 | -0.21 | 6.5×10^-04^ | *TMEM55B* |
|  | Hapmap47512-BTA-114443 | 10 | 27485823 | 0.33 | 0.12 | 2.6×10^-10^ | 0.30 | 0.14 | 0.00221 | / |
| rs43625662 | BTB-00417448 | 10 | 28285995 | 0.34 | 0.11 | 7.2×10^-10^ | 0.33 | 0.24 | 5.0×10^-08^ | / |
|  | BTA-78285-no-rs | 10 | 86717378 | 0.54 | -0.10 | 4.7×10^-09^ | 0.54 | -0.11 | 0.0097 | *NEK9* |
| rs109452965 | ARS-BFGL-NGS-84575 | 26 | 14912765 | 0.38 | 0.10 | 5.3×10^-08^ | 0.39 | -0.02 | 0.632 | *CEP55* |

^a^ Bovine UMD 3.1 genome assembly except for ARS-BFGL-NGS-83544 that was relocated from the USDA-AGIL SNP coordinates

^b^ Estimated coefficient of SNPs (additive effect) from the mixed model

^c^ Bovine UMD 3.1 genome assembly
