# Supplementary material for: Cattle Sex-Specific Recombination and Genetic Control from a Large Pedigree Analysis
Source: PLoS Genet. 2015 Nov 5;11(11):e1005387. doi: 10.1371/journal.pgen.1005387 (PMC4634960; doi:10.1371/journal.pgen.1005387)
Supplement: S5 Table — (DOCX) [file pgen.1005387.s018.docx]

**Table S5. SNPs removed due to suspicious LD patterns near recombination hotspots.** We calculated pairwise LD statistics for the SNPs located close to a recombination hotspot. Pairwise LD was measured between a SNP of interest and all other SNPs on the same chromosome. Those SNPs close to recombination hotspots and showing unexpected LD patterns were removed.

| **SNP** | **Chr** | **Position^a^** | **Hotspot Recombination**  **Rate nearby** | | **LD Pattern** |
| --- | --- | --- | --- | --- | --- |
|  |  |  | **Female** | **Male** |  |
| ARS-BFGL-NGS-105151 | 2 | 104870344 | 0.0461 | 0.0452 | No LD with nearby SNPs but one |
| ARS-BFGL-NGS-21251 | 2 | 105006704 | 0.0461 | 0.0452 | No LD with nearby SNPs but one |
| BTA-102476-no-rs | 6 | 106291585 | 0.00677 | 0.02284 | Max LD far from the SNP |
| ARS-BFGL-NGS-12278 | 6 | 107383105 | 0.00677 | 0.02284 | Max LD far from the SNP |
| BTA-25280-no-rs | 6 | 107437966 | 0.00677 | 0.02284 | Max LD far from the SNP |
| BovineHD0600030859 | 6 | 109661494 | 0.0238 | 0.0322 | Max LD far from the SNP |
| ARS-BFGL-NGS-110156 | 6 | 109868839 | 0.0238 | 0.0322 | Max LD far from the SNP |
| ARS-BFGL-NGS-36431 | 10 | 87411126 | 0.01401 | 0.01889 | No LD with nearby SNPs but one |
| ARS-BFGL-NGS-19018 | 10 | 87731360 | 0.01386 | 0.01845 | No LD with nearby SNPs but one |

^a^ USDA-AGIL SNP coordinates
